# Extracellular vesicle-based therapeutic strategies for spinal tumors and associated nerve damage: advances, challenges, and future directions

**DOI:** 10.3389/fcell.2026.1934352

**Published:** 2026-09-02

**Authors:** Mingxuan Yang, Mengsi Tian, Jie Wang, Zhijian Ma, Rui Chen, Xinyu Ben

**Affiliations:** 1 Department of Neurology, Hainan General Hospital, Hainan Affiliated Hospital of Hainan Medical University & Key Laboratory of Brain Science Research and Transformation in Tropical Environment of Hainan Province, Haikou, Hainan, China; 2 International Center for Aging and Cancer, Hainan Medical University, Hainan Academy of Medical Sciences, Haikou, Hainan, China; 3 The Children Rehabilitation Department, Hainan Women and Children’s Medical Center, Hainan Medical University, Haikou, China; 4 Center of Geriatrics, Hainan Hospital Affiliated to Hainan Medical University, Haikou, Hainan, China

**Keywords:** engineered extracellular vesicles, extracellular vesicles, nerve damage, spinal tumors, therapeutic strategies

## Abstract

The clinical management of spinal tumors presenting with acute spinal cord compression with consequent neural injury poses two concurrent challenges: achieving oncological control and preserving or restoring neurological function. Surgical decompression and chemoradiotherapy can relieve mechanical compression and reduce tumor burden, but they do not directly restore the damaged neural microenvironment. In recent years, extracellular vesicles (EVs) have emerged as promising cell-free nanotherapeutic candidates. Preclinical evidence suggests that they can cross the blood-spinal cord barrier and deliver functional biomacromolecules. This narrative review critically evaluates recent advances in EV-based interventions for spinal tumors and associated neural injury. To improve interpretive rigor, we distinguish evidence according to translational proximity, from human-derived data and orthotopic spinal tumor models to non-spinal tumor and non-neoplastic spinal cord injury models. We discuss how natural and engineered EVs may improve selective targeting, help overcome chemotherapy resistance, regulate neuroinflammation, and promote axonal regeneration. However, EV therapeutics for spinal tumors remain at an early preclinical stage. In spinal microenvironments that may contain residual tumor cells, EVs can exert double-edged effects, including off-target toxicity, pro-tumorigenic niche remodeling, and enhanced immune evasion. Scalable isolation, purification, and standardized potency assessment under international consensus frameworks remain major barriers. Dedicated tumor-bearing spinal models are therefore required to determine whether EV-based interventions can be developed into safe, viable, and personalized therapeutic approaches.

## Graphical Abstract

**Figure:**
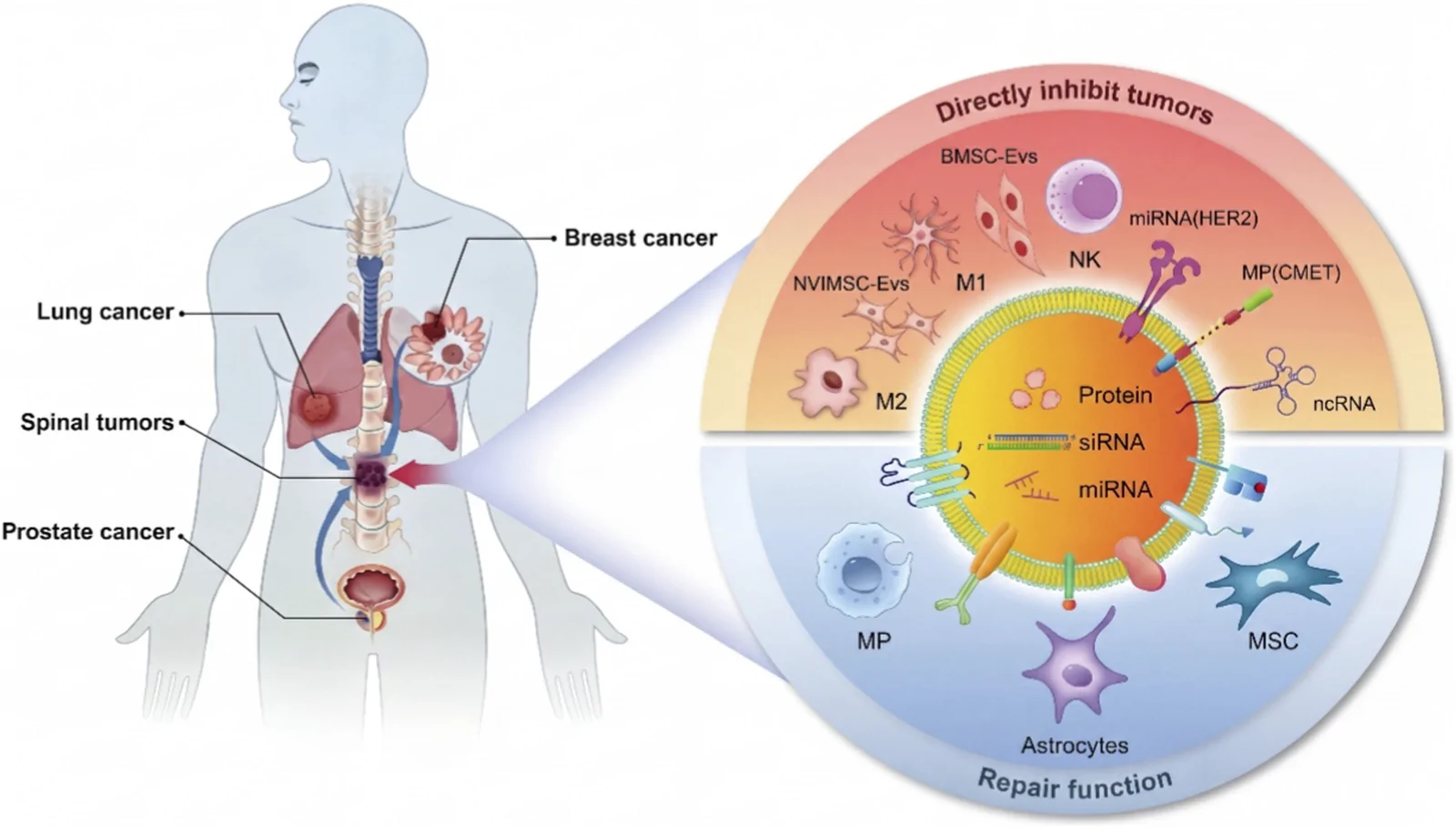

## 1 Introduction

Spinal tumors, encompassing a broad spectrum from benign lesions to highly aggressive malignancies, present a formidable clinical challenge due to the ensuing spinal cord compression and neurological dysfunction. Although anatomically classified into epidural, intradural-extramedullary, and intradural-intramedullary categories (Chamberlain and Tredway, 2011; Kaloostian et al., 2014), primary spinal bone tumors are epidemiologically rare, accounting for only 5% of all primary bone tumors (Kumar et al., 2020). By contrast, the spine is the most frequent site for malignant bone metastases. Approximately 50%–70% of patients with cancer experience spinal involvement during their disease course, with the highest incidences originating from lung, breast, and prostate cancers (Chiu and Mehta, 2020; Coleman et al., 2020). Metastatic spinal tumors (MSTs) not only elicit intractable pain but also predispose patients to vertebral collapse and acute spinal cord compression, thereby severely compromising quality of life (Dang et al., 2015). Given the clinical predominance and complex pathological mechanisms of MSTs, this review focuses on spinal metastases originating from lung, breast, and prostate cancers, alongside the resultant neurological injuries.

Management of spinal tumours must address three linked problems: a space-occupying lesion that may require urgent decompression or cytoreduction, restricted access of systemically administered agents across the blood–spinal cord barrier (BSCB), and persistent neurological dysfunction after tumour control. Surgery remains the most direct means of relieving mass effect and achieving local control, whereas radiotherapy and systemic therapy help treat residual or disseminated disease. These interventions do not by themselves restore injured neural tissue or remodel the postoperative microenvironment; perioperative manipulation may further injure the spinal cord or nerve roots, and established axonal degeneration or demyelination may persist (Dang et al., 2015; Leong et al., 2024; Nater and Fehlings, 2016; El-Hajj et al., 2022). EVs, particularly small EVs (sEVs) with diameters of approximately 30–200 nm, have therefore attracted attention as adjunctive, cell-free delivery and regenerative platforms (Pegtel and Gould, 2019). According to the International Society for Extracellular Vesicles guidelines (MISEV), these endogenous nanoscale vesicles mediate intercellular communication by transferring proteins, lipids, and nucleic acids (Welsh et al., 2024). Compared with viable stem-cell transplantation, acellular EV preparations avoid risks arising from uncontrolled donor-cell proliferation and ectopic engraftment and may have lower immunogenicity; however, this does not imply that all EV products are intrinsically non-tumorigenic. Their biological safety depends on the source cell, endogenous cargo, engineering process, dose, biodistribution, and recipient microenvironment (Liang et al., 2021).

Therapeutic and endogenous EVs must therefore be distinguished. Engineered EVs or EVs derived from carefully characterized non-malignant cells can function as delivery vehicles and may increase local drug exposure while reducing systemic toxicity. By contrast, tumor-derived endogenous EVs can carry oncogenic proteins and nucleic acids, suppress antitumor immunity, and remodel pre-metastatic niches (Welsh et al., 2024; Gong et al., 2025; Javdani-Mallak et al., 2025). Thus, the reduced cell-transplantation risk associated with a defined, acellular EV product is not equivalent to an absence of tumor-promoting activity across all EV sources. This source- and context-dependent duality is especially important in spinal lesions, where residual tumor cells, neural injury, and an immunoregulatory repair milieu may coexist.

Accordingly, this narrative review critically examines recent advances in EV-based therapies for metastatic spinal tumors and associated neurological repair. We assess EV-mediated antitumor delivery, microenvironmental regulation, and neural regeneration while explicitly distinguishing evidence from human data, orthotopic spinal tumor models, non-spinal tumor models, and non-neoplastic SCI models. We also examine manufacturing, biodistribution, and safety constraints that must be resolved before clinical translation.

We conducted a narrative PubMed search covering publications available through early 2026, with emphasis on studies published from 2020 onward. Search terms combined extracellular vesicles with spinal tumors, spinal cord tumors, metastasis, targeted delivery, tumor microenvironment and neural repair. Because no prespecified protocol, formal risk-of-bias assessment or PRISMA workflow was used, the article should be interpreted as a narrative synthesis rather than a systematic review.

## 2 Extracellular vesicle biology, therapeutic advantages, and TA-SCI time windows

### 2.1 Definition and standardized classification of extracellular vesicles

EVs are actively secreted, lipid-bilayer-enclosed particles that mediate intercellular communication and cargo transfer (Pegtel and Gould, 2019). Because vesicle biogenesis cannot usually be established with complete certainty, MISEV 2023 recommends operational descriptors based on characteristics such as particle size, including the term sEVs (Welsh et al., 2024). EV cargoes include proteins, lipids, nucleic acids and glycoconjugates derived from the parent cell (Pegtel and Gould, 2019). In spinal tumors, EVs may originate from tumor cells, mesenchymal stem cells (MSCs), immune cells or neural cells. This heterogeneity underlies their context-dependent effects on the microenvironment.

### 2.2 Therapeutic advantages and scientific risk assessment of EVs

**FIGURE 1:**
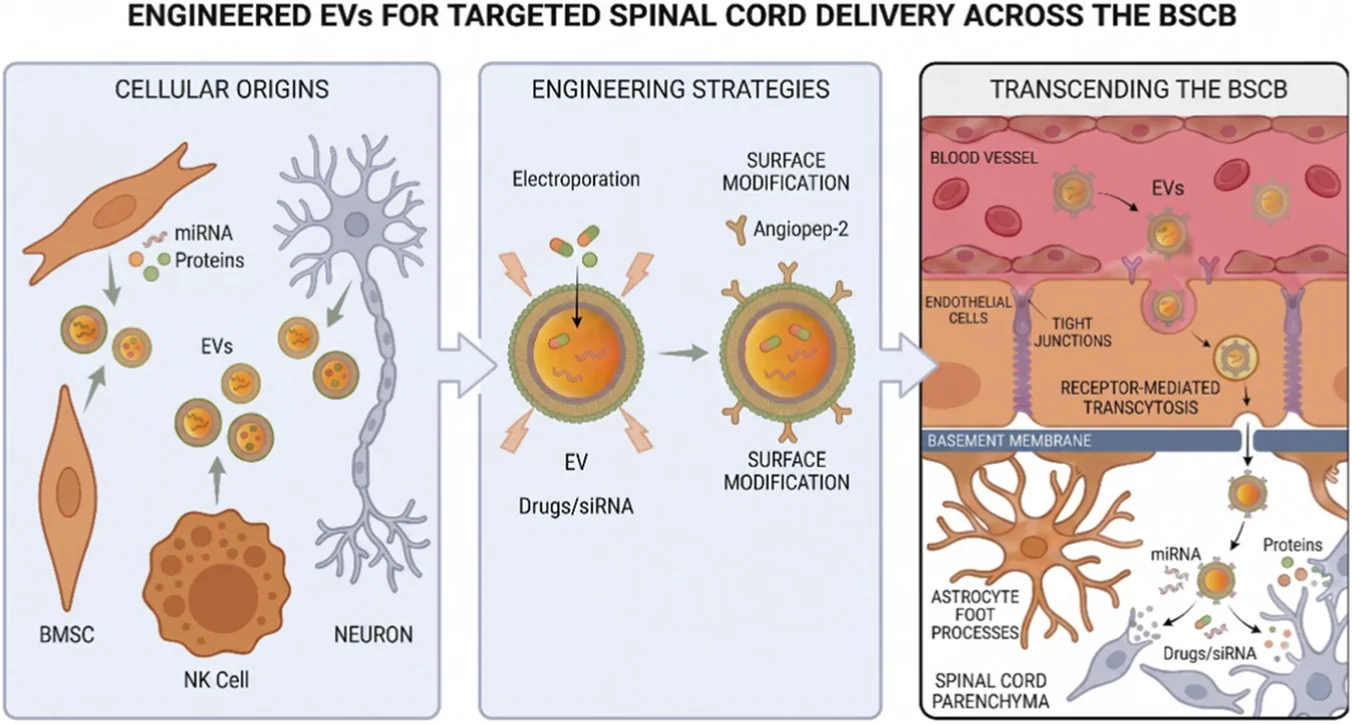
Engineered EVs for targeted spinal cord delivery across the BSCB.

EVs offer three interrelated therapeutic attributes (Figure 1). First, vesicles derived from mesenchymal stromal cells, immune cells, and neural cells can package regulatory RNAs, proteins, and lipids within a membrane-protected carrier. Second, therapeutic cargoes such as drugs or siRNAs can be loaded into EVs, and surface ligands such as Angiopep-2 can be introduced to increase receptor-dependent uptake. Third, the nanoscale membrane structure may facilitate endothelial uptake and transcytosis across a disrupted or ligand-addressable BSCB, followed by cargo release within the spinal cord parenchyma. Together, these properties provide a mechanistic basis for combining lesion-directed antitumor delivery with neural-tissue support. However, Figure 1 represents a therapeutic design framework rather than proof of efficient delivery in spinal tumors: trans-BSCB transport, lesion-to-liver exposure, cargo retention, and receptor specificity remain dependent on EV source, engineering method, dose, and disease state (Liang et al., 2021; Kong et al., 2025).

These proposed advantages are accompanied by source- and context-dependent risks. Most evidence for axonal regeneration and remyelination is derived from non-neoplastic SCI models (Zhang et al., 2022; Hwang et al., 2023), and it remains uncertain whether the same reparative programs are safe when residual spinal tumor cells are present. Pro-angiogenic or anti-apoptotic cargoes could support neural recovery yet also enhance residual-tumor survival, whereas tumor-derived EVs can suppress antitumor immunity and establish pre-metastatic niches (Welsh et al., 2024; Gong et al., 2025; Javdani-Mallak et al., 2025). Accordingly, the translational significance of Figure 1 lies in its modularity: cellular origin, payload, surface ligand, administration route, and treatment phase must be optimized together, with parallel assessment of tumor control, neural repair, and off-target biodistribution.

### 2.3 Pathophysiology and therapeutic time windows of tumor-associated spinal cord injury

Tumor-associated spinal cord injury (TA-SCI) is a composite pathological syndrome that includes chronic tumor-related compression and ischemia, perioperative mechanical or perfusion injury, postoperative inflammation associated with minimal residual disease (MRD), and neural damage caused by radiotherapy or chemotherapy. These processes may overlap and evolve over time, producing distinct therapeutic requirements.

**FIGURE 2:**
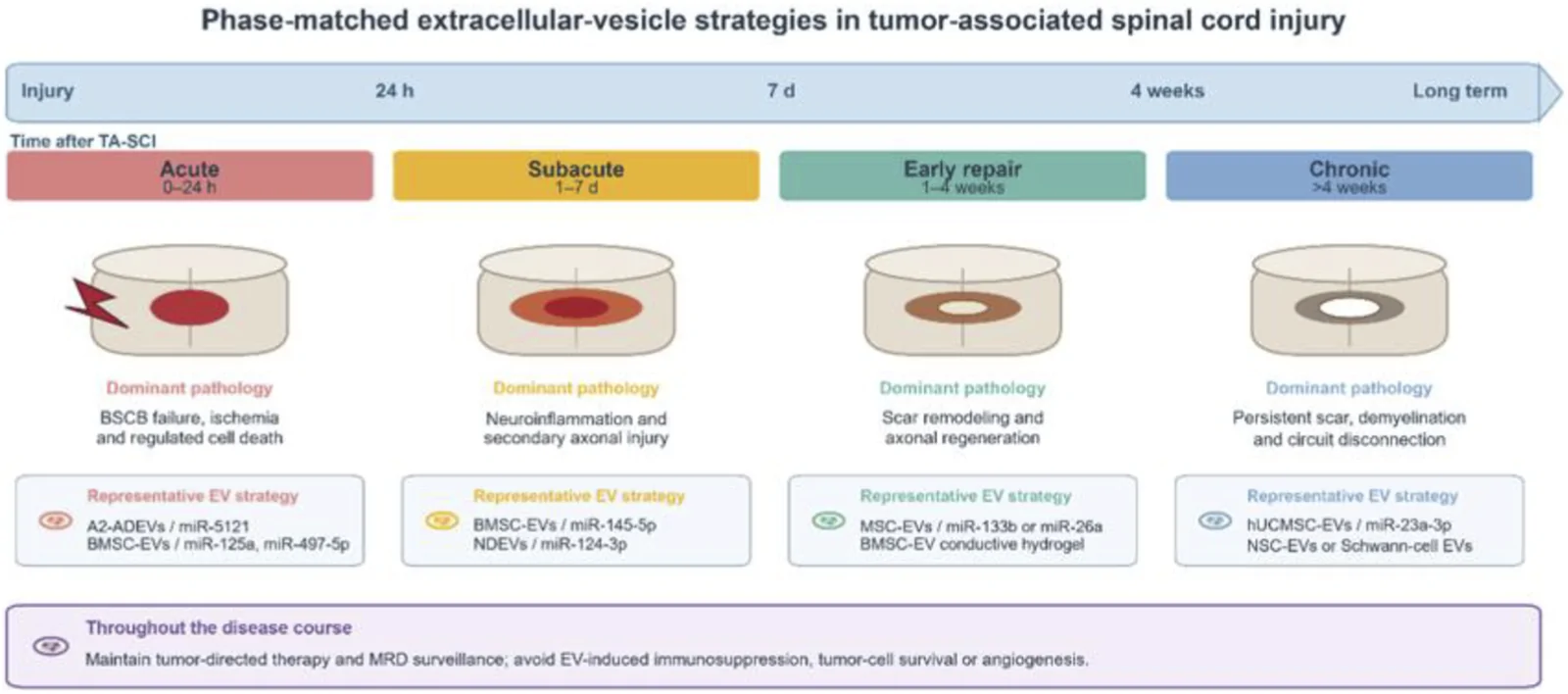
Phase-matched extracellular vesicle strategies for tumor-associated spinal cord injury.

TA-SCI can be operationally divided into four therapeutic windows (Figure 2). During the acute phase (0–24 h), blood–spinal cord barrier disruption, edema, ischemia, oxidative stress, and neuronal or oligodendroglial death predominate. EV-based interventions should therefore prioritize barrier stabilization, antioxidative protection, and inhibition of acute cell death. During the subacute phase (1–7 days), inflammatory-cell infiltration, microglial and macrophage activation, cytokine release, demyelination, and secondary axonal injury become more prominent. EVs may help regulate inflammatory responses, but excessive immunosuppression could impair immune surveillance of residual tumor cells. During the early repair phase (1–4 weeks), glial-scar formation, vascular remodeling, axonal sprouting, and remyelination coexist. Regenerative EVs should therefore be considered only after residual disease and tumor-associated angiogenic risk have been assessed. Beyond 4 weeks, chronic compression, fibrosis, chondroitin-sulfate-proteoglycan accumulation, persistent inflammation, and treatment-related tissue damage may predominate. This stage may require scar remodeling, remyelination, and sustained local delivery strategies (Sintakova and Romanyuk, 2024; Zaki et al., 2023; Romanelli et al., 2022; Tanak et al., 2025; Bach et al., 2026; Millesi et al., 2026).

This phase-matched framework highlights a central limitation of the field: most EV studies use non-neoplastic SCI models rather than tumour-bearing TA-SCI models. Effects observed in conventional SCI should therefore not be treated as direct evidence of efficacy or safety in spinal tumours. The therapeutic implications are considered according to this temporal sequence, with acute and subacute inflammation and cell death distinguished from later axonal, vascular and myelin repair. Future studies should integrate tumour burden, compression severity, decompression, MRD, treatment exposure, neurological outcomes, EV biodistribution and oncological safety within the same model.

## 3 Multidimensional roles of extracellular vesicles in suppressing spinal tumors

More than 70% of clinically encountered spinal tumors are metastatic, most commonly arising from breast, lung and prostate cancers. Consequently, much of the available EV evidence comes from *in vivo* models of these primary malignancies rather than from spinal lesions. EV interventions directed at the spinal bone-nerve compression interface remain exploratory.

**FIGURE 3:**
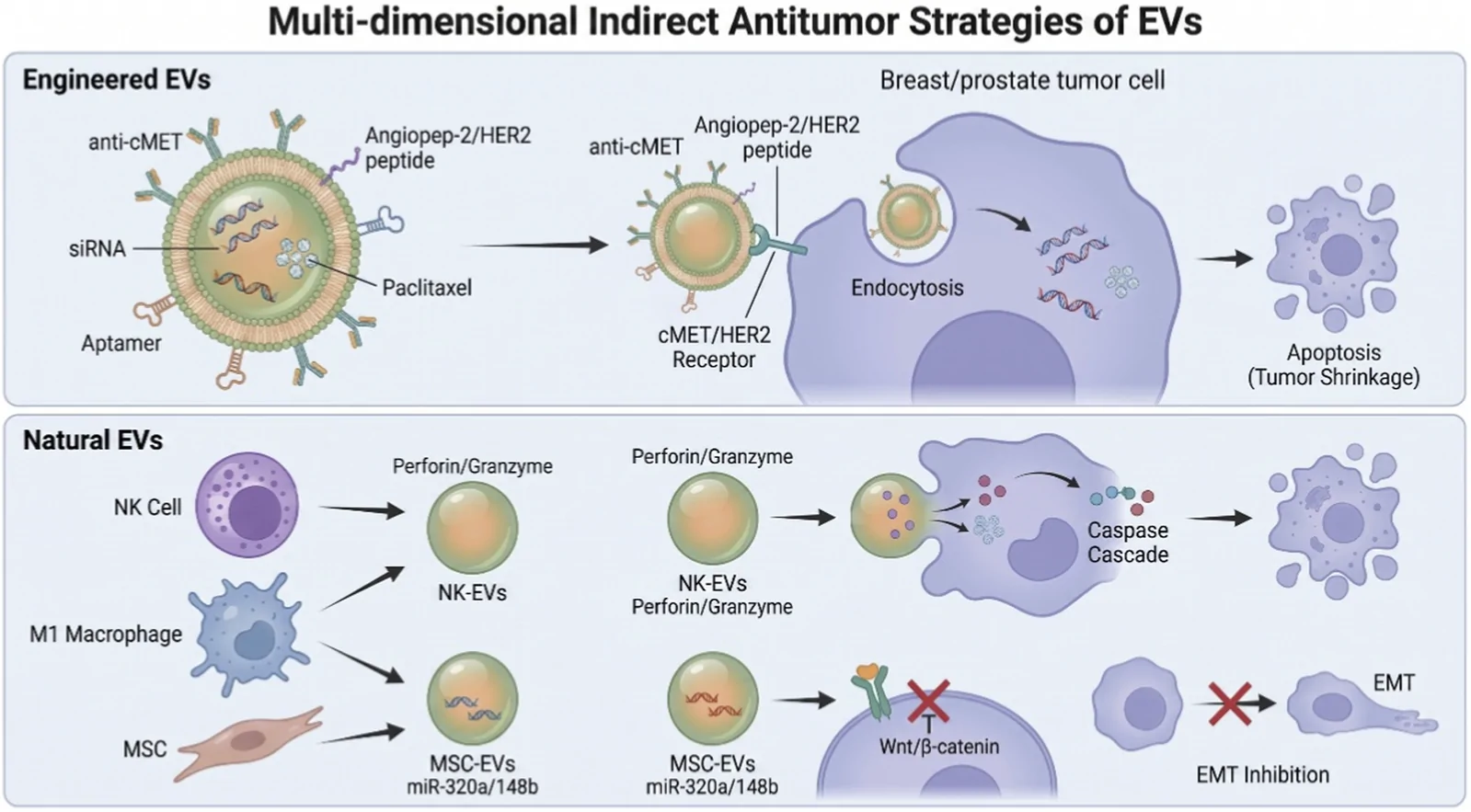
Multi-dimensional indirect antitumor strategies of EVs.

This section is organized around the two mechanistic branches shown in Figure 3: engineered or modified EVs, including surface-targeted and cargo-loaded systems, and naturally derived EVs, including immune-cell- and MSC-derived vesicles. Within each branch, the available evidence is ranked according to its translational proximity to spinal tumours. Level 1 comprises human-derived or clinical data, whereas Level 2 comprises orthotopic spinal-tumour models. Level 3 includes non-spinal orthotopic tumour models, systemic or organ-specific metastatic models, osseous tumour models and peripheral nerve-sheath tumour models. These models reproduce selected features of metastatic dissemination, bone involvement or neural-tumour biology, but they do not recreate tumour growth at the vertebral–spinal-cord interface. Level 4, non-tumor SCI or related neural-injury models; and Level 5 refers to subcutaneous tumor models, including subcutaneous xenografts or syngeneic tumors, which provide antitumor efficacy information but have the lowest anatomical relevance to spinal tumors. Evidence level and EV origin are independent axes: the former indicates proximity to human spinal tumors, whereas the latter follows the mechanistic organization of Figure 3.

Suppressing the primary source of metastasis and highly aggressive tumor clones may reduce subsequent spinal dissemination. The next two subsections compare antitumour evidence from engineered and naturally derived EVs within this evidence hierarchy. The antitumor actions of engineered and naturally derived EVs are summarized in Figure 3.

### 3.1 Engineered or modified extracellular vesicles

Surface engineering can increase EV uptake by malignancies with a high propensity for bone or spinal metastasis, although spine-specific targeting remains unproven.

#### 3.1.1 Level 1: Human-derived or clinical evidence

In patients with metastatic pancreatic ductal adenocarcinoma, mesenchymal stromal cell-derived exosomes electroporated with KRASG12D-specific siRNA achieved first-in-human clinical feasibility, acceptable safety, pharmacodynamic target engagement, and preliminary disease stabilization (Kalluri et al., 2025). This is Level 1 evidence for a siRNA-loaded engineered EV platform, but it does not demonstrate efficacy against spinal tumors or spinal metastases.

#### 3.1.2 Level 2: Orthotopic spinal tumor models

In a high-grade spinal cord glioma model in Göttingen minipigs, spatially heterogeneous hypoxia, proliferation, inflammation, neovascularization, and oxidative stress were reproduced (Tora et al., 2023). In a separate intramedullary 9 L gliosarcoma rat model, blood-spinal cord barrier manipulation, tumor burden, and neurological outcomes could be evaluated (Bhimreddy et al., 2026). These are anatomically relevant Level 2 platforms, but neither has yet been used to test an engineered EV antitumor intervention. Level 2 therefore remains an evidence gap rather than a category that can be filled by non-spinal or non-tumor models. Orthotopic and genetically engineered spinal tumor models should be prioritized for EV gain- and loss-of-function experiments (Pando et al., 2026).

#### 3.1.3 Level 3: Xenograft, metastatic, osseous, and peripheral nerve-sheath tumor models

In orthotopic mammary MDA-MB-231 xenografts, macrophage-derived EV membranes coated doxorubicin-loaded PLGA nanoparticles and were functionalized with a c-Met-binding peptide through DSPE-PEG2000 lipid insertion (Li et al., 2020). EV coating increased intracellular doxorubicin accumulation 3.31-fold relative to PLGA nanoparticles. c-Met functionalization produced an additional approximately twofold increase relative to the non-targeted EV-coated formulation. The corresponding apoptotic fractions were 39.73% and 29.27%, respectively. *In vivo* tumor accumulation was 1.62-fold higher than that of the non-targeted hybrid and 2.22-fold higher than that of PLGA nanoparticles. These values quantify biological targeting rather than peptide-insertion efficiency; ligand density, membrane retention, batch comparability and scale-up performance were not reported.

The orthotopic mammary xenograft model provides Level 3 evidence, but its breast-tumor location does not reproduce spinal bone, neural compression, or the spinal tumor microenvironment.

In a Lewis lung carcinoma pulmonary-metastasis model, paclitaxel-loaded EVs produced more than 50-fold greater cytotoxicity than free paclitaxel (Kim et al., 2016). This Level 3 model supports EV-mediated drug delivery against metastatic disease, but it does not reproduce a spinal lesion. The approximately 10% loading efficiency and the absence of reported batch-to-batch reproducibility also limit translation toward GMP-scale manufacturing.

Because the *in vivo* model was pulmonary metastatic Lewis lung carcinoma rather than a spinal lesion or a subcutaneous primary tumor, this evidence is classified as Level 3.

In mice bearing HEI-193 schwannoma cells implanted in the sciatic nerve, genetically engineered microvesicles carrying cytosine deaminase–uracil phosphoribosyltransferase suicide mRNA and protein, combined with systemic 5-fluorocytosine, inhibited and regressed tumors (Mizrak et al., 2013). This is Level 3 peripheral nerve-sheath evidence. The model does not contain the spinal canal, vertebral compartment, spinal cord parenchyma, or tumor-associated blood-spinal cord barrier, and therefore cannot be upgraded to Level 2.

In primary bone-tumor models, EV-mediated microenvironmental remodeling has also been linked to tumor suppression through emerging mechanisms such as ferroptosis.

In osteosarcoma models, hydroxyapatite-affinity peptide SDSSD-modified BMSC exosomes loaded with capreomycin accumulated in osseous tumors and induced Keap1/Nrf2/GPX4-associated ferroptosis (Chen W. et al., 2023). This is Level 3 osseous xenograft evidence that supports bone-directed delivery, but it does not establish efficacy in a vertebral or intramedullary spinal tumor.

#### 3.1.4 Level 4: Non-tumor spinal cord injury models

Compared to standard primary tumor targeting, the direct delivery of EVs into the spinal milieu poses a distinct challenge, largely impeded by the bone matrix, mechanical neural compression, and the blood-spinal cord barrier (BSCB). Currently, research in this domain is predominantly confined to exploratory laboratory stages.

In a non-neoplastic spinal cord injury model, Angiopep-2 (Ang2) was displayed on EVs through biological engineering of M2 microglia rather than post-isolation chemical conjugation. The engineered EVs showed LRP1-associated enrichment within injured spinal cord parenchyma (Kong et al., 2025). However, the study did not report the proportion of Ang2-positive EVs, ligand molecules per EV, batch-to-batch variability or scale-up yield. It therefore provides Level 4 evidence for relative barrier crossing and lesion targeting, but not a standardized coupling-efficiency estimate or antitumour efficacy in a spinal tumour.

In a spinal cord contusion model, peptide-painted autologous plasma exosomes carrying neuron-targeting and growth-facilitating peptides promoted axonal regrowth, vascular formation, reconstruction of intraspinal circuits, and functional recovery (Ran et al., 2023). This is Level 4 evidence because the model was non-neoplastic spinal cord injury. It supports delivery and neural-repair feasibility, but not EV efficacy against spinal tumors.

These findings support the feasibility of barrier-crossing delivery, but their antitumor efficacy in true spinal tumor lesions still requires direct validation.

#### 3.1.5 Level 5: Subcutaneous primary-tumor models

In subcutaneous SK-OV-3 xenografts, HER2 dual-targeted EVs were generated from stable HEK-293 producer cells expressing a C1C2-anchored HER2-binding peptide and miR-HER2-E1 (Wang et al., 2020). This platform therefore combines genetic surface display with endogenous miRNA loading rather than post-isolation peptide conjugation. HER2 binding was confirmed by ELISA, uptake was enhanced in HER2-positive cells, and the delivered miRNA suppressed HER2 synthesis and reduced tumour volume *in vivo*.

Particle-size distribution and EV markers were retained, supporting basic product identity. However, peptide copies per EV, the proportion of ligand-positive EVs, formal batch comparability, *in vivo* ligand stability and scale-up performance were not reported. Similar tumour suppression at 3 and 30 μg per mouse suggests a plateau in biological response, but does not define coupling efficiency. The subcutaneous SK-OV-3 model remains Level 5 because it does not reproduce the vertebral bone, marrow or neural microenvironment.

The *in vivo* experiment used a subcutaneous SK-OV-3 xenograft and is therefore classified as Level 5; it was not a breast-cancer spinal-metastasis model.

In prostate-cancer models, anti-PSMA exosome mimetics were generated by nucleofection or lentiviral engineering of U937 cells to display the peptide WQPDTAHHWATL, followed by serial extrusion and size-exclusion chromatography (Duan et al., 2025; Severic et al., 2021; Xu et al., 2026; Zhang et al., 2023). Targeted and control mimetics showed comparable hydrodynamic diameters, polydispersity indices below 0.2 and zeta potentials of approximately −18 mV. Nanoparticle-tracking analysis showed mean diameters of 133–135 nm and particle concentrations of 2.16–2.49 × 10^10 particles/mL. Spherical morphology and retention of LFA-1, Moesin, TSG101 and CD63 supported membrane and protein integrity after extrusion. These findings indicate preserved particle identity, but they do not establish per-vesicle ligand density or manufacturing reproducibility.

PSMA-positive LNCaP and C4-2 B cells showed increased uptake, and accumulation in subcutaneous C4-2 B tumours was significantly higher than that of non-targeted mimetics (Severic et al., 2021). Nevertheless, per-vesicle peptide density, the magnitude of the tumour-accumulation gain and batch-to-batch variation were not reported. Extrusion may improve yield and scalability relative to natural EV secretion, but stable producer-cell control, repeated extrusion and chromatographic purification remain sources of manufacturing variability. The model is Level 5; penetration into vertebral osteoblastic metastases and protection of spinal nerves or marrow have not been demonstrated.

In subcutaneous 4T1 breast-tumor models, paclitaxel- or docetaxel-loaded M1 macrophage-derived exosomes increased antitumor activity (Zhao et al., 2022; Wang et al., 2019). Because the vesicles were exogenously drug-loaded, they are classified here as engineered systems even though their carrier source was an M1 macrophage. These studies provide Level 5 evidence.

#### 3.1.6 Supporting bone-targeting evidence outside levels 1–5

Recent engineering studies over the past 5 years (2021–2025) have begun to improve EV access to bone- and spinal lesion-related microenvironments.

In murine bone-loss models, CXCR4-positive mammalian exosomes selectively accumulated in bone marrow (Hu et al., 2021). In a separate osteoporosis model, synthetic-biology bacterial EVs co-displaying BMP-2 and CXCR4 achieved *in vivo* bone targeting (Liu H. et al., 2024). Both are delivery-feasibility studies in non-tumor bone-loss settings, so they do not satisfy Levels 1–5 of the present antitumor hierarchy or establish efficacy in spinal tumors.

### 3.2 Naturally derived extracellular vesicles

Naturally derived EVs may influence dissemination to the spine by altering tumor-cell behavior, antitumor immunity and the metastatic microenvironment.

#### 3.2.1 Level 1: Human-derived or clinical evidence

In adults with intradural spinal tumors, including intraspinal ependymoma, meningioma, hemangioma, and schwannian tumors, cerebrospinal-fluid EV subpopulations distinguished tumor entities and ependymoma grades (Salviano-Silva et al., 2025). This is Level 1 human spinal-tumor EV evidence, but it is diagnostic and pathobiological rather than therapeutic.

In breast-cancer bone-tropism models, tumor cells released miR-19a-enriched EVs that homed to bone, induced osteoclastogenesis, disrupted osteogenic homeostasis, and generated an osteolytic niche for cancer-cell colonization (Wu et al., 2021).

Human breast-tumor specimens and circulating exosomal miR-19a provide a Level 1 pathobiological component, whereas the accompanying preclinical osseous-metastasis experiments provide Level 3 evidence. The EV homing is bone-tropic rather than spine-specific, and the effect promotes metastatic risk rather than tumor suppression.

#### 3.2.2 Level 2: Orthotopic spinal tumor models

No identified PubMed-indexed original study has tested naturally derived EVs as an antitumor therapy in an orthotopic spinal tumor model. The minipig spinal cord glioma and rat intramedullary gliosarcoma platforms described above (Tora et al., 2023; Bhimreddy et al., 2026) could evaluate both therapeutic efficacy and the risk that regenerative or pro-angiogenic EV cargoes support residual tumor growth, but such experiments remain unavailable. Naturally derived EVs therefore also lack direct Level 2 therapeutic evidence.

#### 3.2.3 Level 3: Xenograft and metastatic tumor models

In cancer-cell models, NK-cell-derived EVs containing perforin and granzymes activated caspase-dependent apoptosis (Wu et al., 2019). In an orthotopic hepatocellular-carcinoma xenograft, unmodified human NK-cell exosomes containing perforin, granzyme B, FasL, and TRAIL inhibited tumor growth and activated caspase-dependent apoptosis; a parallel subcutaneous arm showed the same antitumor direction (Kim et al., 2022). The orthotopic xenograft provides Level 3 evidence and the subcutaneous arm provides Level 5 evidence, but neither model is spinal.

The *in vitro* cancer-cell observations in (Wu et al., 2019) remain outside Levels 1–5 because they lack an *in vivo* model. The orthotopic and subcutaneous HCC results in (Kim et al., 2022) strengthen the naturally derived NK-EV branch in Figure 3, but they remain extrapolations from tumor models.

These potential benefits must be considered alongside the source-dependent capacity of EVs to promote tumor growth and metastatic-niche formation.

In osseous-metastasis experiments, naturally secreted tumor EVs promoted bone colonization rather than tumor suppression (Wu et al., 2021). This Level 3 risk evidence should be considered alongside therapeutic EV studies because it shows that EVs can also remodel the metastatic niche in an oncogenic direction.

#### 3.2.4 Level 4: Non-tumor spinal cord injury models

In an experimental spinal cord injury model, naturally derived placental MSC exosomes improved locomotor and bladder function and activated endogenous neural stem/progenitor cells (Zhou et al., 2021). This is Level 4 spinal delivery and regenerative evidence, not antitumor evidence. Regenerative signaling must be tested for oncological safety in a tumor-bearing Level 2 model before translation to postoperative spinal-tumor settings.

#### 3.2.5 Level 5: Subcutaneous primary-tumor models

In 2D triple-negative breast-cancer cell models, M1-polarized macrophage-derived EVs exerted pro-apoptotic effects. Because these experiments lacked an *in vivo* tumor model and pharmacokinetic assessment, they remain outside the five-level hierarchy and require validation *in vivo*.

In a subcutaneous 4T1 tumor-bearing mouse model, unmodified M1 exosomes produced antitumor activity, whereas paclitaxel loading further increased efficacy (Wang et al., 2019). The unmodified M1-exosome arm is Level 5 naturally derived EV evidence; the paclitaxel-loaded arm is Level 5 engineered EV evidence. These models do not reproduce spinal metastatic disease.

In non-small-cell lung-cancer models, BMSC-secreted EVs transferred let-7i, downregulated KDM3A, and induced cell-cycle arrest in tumor cells (Liu et al., 2021). The mechanism is relevant to suppression of tumor dissemination toward bone and spine.

The *in vivo* evidence for this mechanism came from a subcutaneous A549 tumor model and is therefore classified as Level 5.

In lung- and breast-cancer models, human umbilical-cord MSC-derived EVs were reported to inhibit EMT and Wnt/β-catenin signaling through delivery of miR-320a and miR-148b, thereby reducing tumor-cell motility (Xie and Wang, 2022).

The miR-320a/SOX4 mechanism is supported by a subcutaneous lung-cancer model and is classified as Level 5 (Xie and Wang, 2022). The miR-148 b claim is not counted as positive Level 1–5 evidence because the primary breast-cancer article was retracted for unresolved data-integrity and raw-data concerns (Frontiers Editorial Office, 2024).

In complex microenvironments, MSC-derived EVs can have opposing effects (Easwaran et al., 2025). After spinal-tumor surgery, pro-angiogenic cargoes such as VEGF could support residual tumor clones, local recurrence, or drug resistance within an ischemic and inflammatory wound environment (Papadopoulos et al., 2025).

Tumor-derived exosomes can educate bone-marrow MSCs toward a tumor-promoting phenotype in tumor models (Lin et al., 2016), providing Level 3 risk evidence. Conversely, menstrual MSC exosomes suppressed VEGF-associated angiogenesis and tumor growth in a subcutaneous prostate-cancer model, whereas bone-marrow MSC exosomes produced an opposing angiogenic response (Alcayaga-Miranda et al., 2016). These Level 5 data support a source- and context-dependent double-edged-sword interpretation, but they do not prove postoperative spinal-tumor recurrence.

Therefore, these tumor-suppressive mechanisms, primarily delineated in subcutaneous models, necessitate rigorous safety validation within the authentic bone-nerve-tumor spinal microenvironment.

### 3.3 Cross-level synthesis and translational priorities

Figure 3 integrates the two EV-origin branches with the five-level evidence hierarchy. The branch structure describes the biological source and mode of intervention, whereas the evidence level indicates how closely each model approximates human spinal-tumour disease. Level 1 currently contains human feasibility, diagnostic, and pathobiological data but no therapeutic validation in spinal tumors. Level 2 remains the decisive evidence gap for both EV categories. Most antitumor efficacy is supported by Level 3 and Level 5 models, whereas Level 4 supports spinal delivery or neural repair only in non-neoplastic injury.

Across evidence levels, current surface-engineering studies cannot be compared quantitatively because genetic display, covalent conjugation, lipid insertion and non-covalent modification have been evaluated using non-equivalent endpoints. HER2, PSMA, Ang2 and c-Met studies mainly report receptor-dependent uptake or lesion accumulation. By contrast, per-EV ligand density, the proportion of modified EVs, membrane integrity after engineering, batch-to-batch variability and scale-up recovery are seldom reported together. Table 3 therefore summarizes the available engineering approaches while distinguishing reported targeting performance from unreported coupling efficiency and manufacturing readiness.

Future intervention studies in tumor-bearing spinal models should determine whether pro-angiogenic cargoes such as VEGF promote residual-tumor growth. Figure 3 integrates the principal antitumor strategies and their translational evidence levels, whereas the therapeutic parameters of EVs from different cellular sources are summarized in Table 1.

**TABLE 1:** Therapeutic, delivery and safety evidence of extracellular vesicles from different sources in spinal-tumor-related models.

Source (cell type)	Cargo	Target/pathway	Tumor-suppressive mechanism	Model context	Spinal disease relevance	Evidence level	Main limitation	References
Engineered MSC exosomes	KRASG12D-specific siRNA	KRASG12D	Clinical feasibility, target engagement and preliminary disease stabilization	Patients with metastatic pancreatic ductal adenocarcinoma	Level 1 clinical benchmark; no spinal-tumor efficacy	Level 1	Not tested in spinal tumors; safety and efficacy in spinal metastases remain unknown	(Kalluri et al., 2025)
Engineered EVs	HER2-affinity peptide + miRNA	HER2	Targeted uptake and HER2 gene silencing	Subcutaneous SK-OV-3 xenograft mouse model	Level 5 extrapolation to HER2-positive spinal metastasis	Level 5	The model does not reproduce the spinal bone microenvironment; peptide stability and in vivo half-life were not established	(Wang et al., 2020)
Engineered macrophage-derived EVs	Anti-cMET scFv + PLGA nanoparticles	cMET	Apoptosis induction and restriction of metastatic capacity	Orthotopic MDA-MB-231 mammary xenograft	Level 3 non-spinal orthotopic evidence	Level 3	The model does not reproduce the spinal bone–nerve–tumor microenvironment	(Li et al., 2020)
Engineered EVs	Paclitaxel	Tumor-cell cytotoxicity	Enhanced drug delivery and cytotoxicity	Pulmonary metastatic Lewis lung carcinoma model	Level 3 metastatic-tumor evidence	Level 3	Approximately 10% loading efficiency; batch reproducibility and GMP-scale manufacturing were not established	(Kim et al., 2016)
Engineered microvesicles	Cytosine deaminase–uracil phosphoribosyltransferase suicide mRNA/protein	Suicide-gene pathway	Tumor inhibition and regression after systemic 5-fluorocytosine	HEI-193 schwannoma cells implanted in the mouse sciatic nerve	Level 3 peripheral nerve-sheath evidence	Level 3	The model lacks the spinal canal, vertebral compartment, spinal cord parenchyma and tumor-associated BSCB	(Mizrak et al., 2013)
Engineered EVs	Anti-PSMA aptamer or peptide + drug/nucleic-acid cargo	PSMA	Receptor-mediated uptake and targeted intracellular release	Subcutaneous prostate-tumor models; bone-metastatic application proposed	Level 5 therapeutic-model evidence; spinal application remains untested	Level 5	Vertebral penetration and protection of spinal nerves or marrow have not been demonstrated	(Duan et al., 2025; Severic et al., 2021; Xu et al., 2026; Zhang et al., 2023)
Engineered M1 macrophage-derived exosomes	Paclitaxel or docetaxel	Tumor-cell apoptosis and macrophage activation	Chemoimmunotherapy and enhanced antitumor activity	Subcutaneous 4T1 breast-tumor models	Level 5 evidence	Level 5	The models do not reproduce spinal metastatic disease; pharmacokinetics and manufacturing comparability remain limited	(Zhao et al., 2022; Wang et al., 2019)
Engineered BMSC exosomes	SDSSD peptide + capreomycin	Keap1/Nrf2/GPX4	Bone accumulation and ferroptosis induction	Osseous osteosarcoma xenograft models	Level 3 osseous-tumor evidence	Level 3	Bone targeting was not validated in vertebral or intramedullary spinal tumors	(Chen W et al., 2023)
Engineered EVs	Angiopep-2-modified cargo	Blood-spinal cord barrier transport	Barrier crossing and cargo delivery into injured spinal parenchyma	Preclinical spinal cord compression and injury models	Level 4 delivery evidence, not antitumor evidence	Level 4	The models are non-neoplastic and do not establish tumor control or oncological safety	(Kong et al., 2025)
Autologous plasma exosomes	Neuron-targeting and growth-facilitating peptides	Neural repair pathways	Axonal regrowth, vascular formation and functional recovery	Spinal cord contusion model	Level 4 neural-repair evidence, not antitumor evidence	Level 4	Spinal-tumor biodistribution, tumor control and recurrence risk remain untested	(Ran et al., 2023)
CXCR4-positive mammalian exosomes	Endogenous bone-targeting cargo	CXCR4-mediated bone homing	Selective accumulation in bone marrow	Murine bone-loss model	Delivery-feasibility evidence outside the antitumor hierarchy	Outside Levels 1–5	No tumor-bearing spinal model or antitumor endpoint was used	(Hu et al., 2021)
Engineered bacterial EVs	BMP-2 + CXCR4	Bone targeting and osteogenic signaling	Bone localization in vivo	Osteoporosis model	Delivery-feasibility evidence outside the antitumor hierarchy	Outside Levels 1–5	No tumor-bearing spinal model or antitumor endpoint was used	(Liu H et al., 2024)
Naturally derived NK-cell EVs	Perforin and granzymes	Caspase-dependent apoptosis	Direct cytotoxic killing of cancer cells	In vitro cancer-cell models	Mechanistic evidence outside the in vivo hierarchy	Outside Levels 1–5	No in vivo pharmacokinetic, biodistribution or spinal-tumor validation	(Wu et al., 2019)
Naturally derived NK-cell exosomes	Perforin, granzyme B, FasL and TRAIL	Caspase-dependent apoptosis	Tumor-growth inhibition and apoptosis activation	Orthotopic and subcutaneous hepatocellular-carcinoma xenografts	Level 3 orthotopic non-spinal evidence; Level 5 subcutaneous evidence	Level 3/Level 5	The models are non-spinal and do not reproduce vertebral or intramedullary disease	(Kim et al., 2022)
Naturally derived M1 macrophage exosomes	Endogenous immunomodulatory cargo	Macrophage-mediated inflammation	Antitumor activity in vivo	Subcutaneous 4T1 breast-tumor model	Level 5 naturally derived EV evidence	Level 5	The model does not reproduce spinal metastatic disease	(Wang et al., 2019)
Naturally derived BMSC EVs	let-7i	KDM3A	Cell-cycle arrest and reduced tumor-cell migration	Subcutaneous A549 lung-cancer model	Level 5 extrapolation to bone or spinal dissemination	Level 5	The model does not reproduce the spinal bone microenvironment	(Liu et al., 2021)
Naturally derived hUCMSC EVs	miR-320a	SOX4 and Wnt/β-catenin	EMT inhibition and reduced tumor-cell motility	Subcutaneous lung-cancer model	Level 5 evidence	Level 5	Long-term safety and efficacy in spinal lesions remain unverified	(Xie and Wang, 2022)
Naturally derived hUCMSC EVs	miR-148 b	Wnt/β-catenin-related EMT	Claimed EMT inhibition and reduced migration	Breast-cancer model reported in a retracted primary article	Not counted as positive Level 1–5 evidence	Excluded	The primary source was retracted for unresolved data-integrity and raw-data concerns	(Frontiers Editorial Office, 2024)
Naturally derived placental MSC exosomes	Endogenous regenerative cargo	Neural stem/progenitor-cell activation	Locomotor and bladder-function recovery	Experimental spinal cord injury model	Level 4 regenerative evidence, not antitumor evidence	Level 4	Regenerative signaling requires oncological-safety testing in a tumor-bearing spinal model	(Zhou et al., 2021)
Naturally derived tumor EVs	miR-19a	Osteoclastogenesis and osteogenic imbalance	Not tumor-suppressive; promotes osteolytic niche formation	Human specimens plus preclinical osseous-metastasis models	Level 1 pathobiological component and Level 3 metastatic-risk evidence	Level 1/Level 3 risk	Bone-tropic rather than spine-specific; demonstrates metastatic risk rather than therapy	(Wu et al., 2021)
Tumor-derived exosomes	Tumor-promoting cargo	Bone-marrow MSC education	Not tumor-suppressive; induces a tumor-promoting MSC phenotype	Tumor-cell/BMSC co-culture and tumor models	Level 3 risk evidence	Level 3 risk	Source- and context-dependent effects require validation in tumor-bearing spinal models	(Lin et al., 2016)
Menstrual MSC exosomes	Endogenous anti-angiogenic cargo	Reactive oxygen species and VEGF-associated angiogenesis	Reduced angiogenesis and prostate-tumor growth	Subcutaneous prostate-cancer model	Level 5 source-dependent evidence	Level 5	The opposing response of bone-marrow MSC exosomes highlights source dependence; spinal safety remains unknown	(Alcayaga-Miranda et al., 2016)
Human CSF EVs	Tumor-associated EV signatures	Tumor classification and ependymoma grading	Diagnostic discrimination rather than tumor suppression	Patients with intradural spinal tumors	Level 1 human spinal-tumor EV evidence	Level 1 diagnostic	No therapeutic intervention or antitumor endpoint was evaluated	(Salviano-Silva et al., 2025)
EV interventions (evidence gap)	—	Orthotopic spinal tumor biology	No direct EV antitumor efficacy has been established	Orthotopic vertebral, epidural, intradural or intramedullary spinal-tumor models remain unteste d with EV therapy	Direct Level 2 evidence gap	Level 2	Model platforms exist, but EV biodistribution, cargo release, tumor control, neurological outcomes and oncological safety require direct testing	(Tora et al., 2023; Bhimreddy et al., 2026; Pando et al., 2026)

### 3.4 Systems-level organization of the spinal tumour microenvironment and EV-mediated bone–nerve crosstalk

**FIGURE 4:**
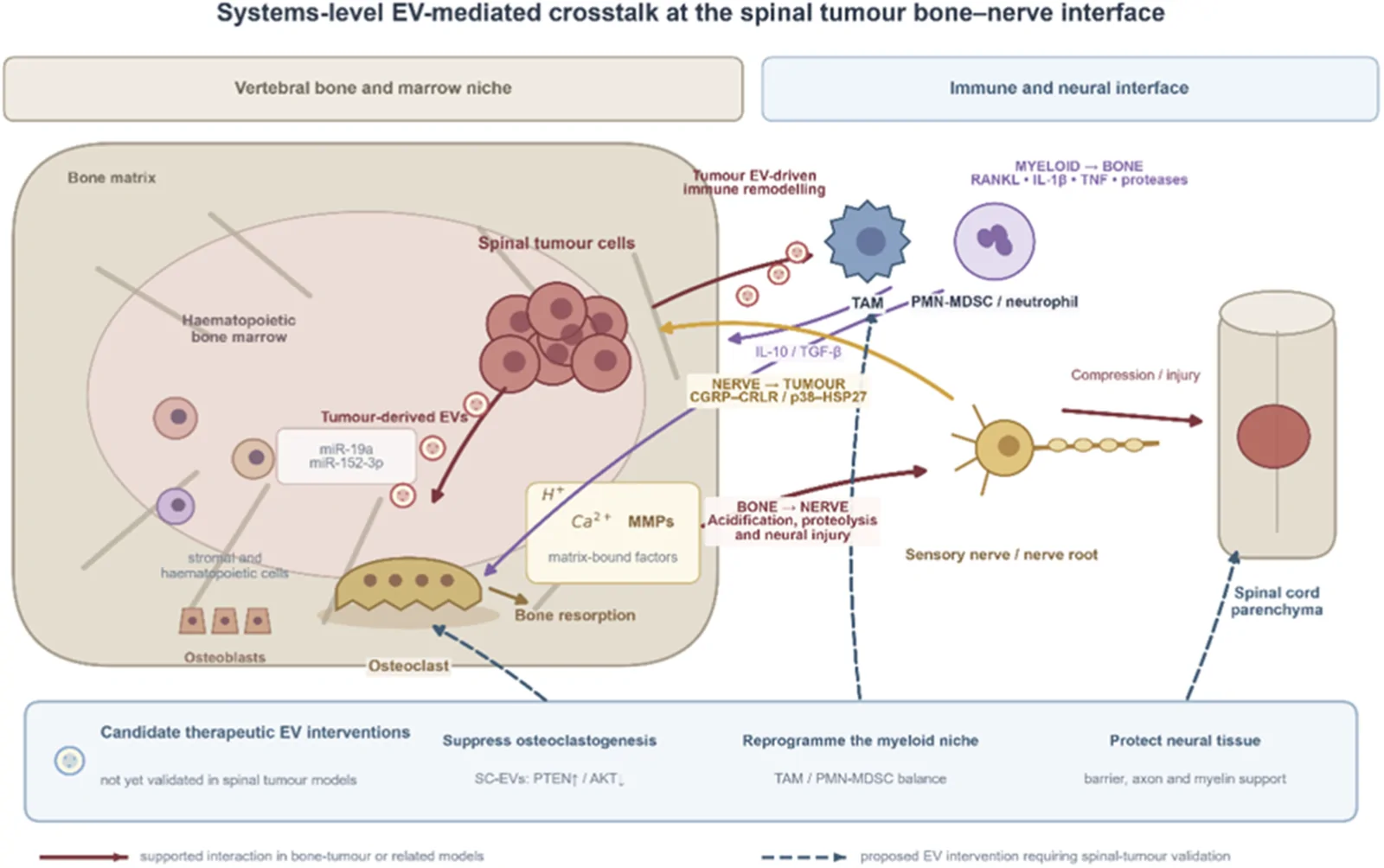
Systems-level extracellular vesicle-mediated crosstalk at the spinal tumor bone–nerve interface.

The spinal tumour microenvironment is an anatomically integrated bone–marrow–immune–neural ecosystem rather than a collection of independently acting cellular components. Within this ecosystem, tumour cells interact with the mineralized bone matrix, osteoclasts, osteoblasts, bone-marrow stromal and haematopoietic cells, endothelial cells, tumour-associated macrophages (TAMs), polymorphonuclear myeloid-derived suppressor cells (PMN-MDSCs) and neutrophils. These interactions extend to adjacent nerve roots and the spinal cord parenchyma, thereby linking tumour progression, bone remodelling, immune regulation and neurological injury (Figure 4) (Clézardin et al., 2021). Tumour-derived cytokines and EV cargoes may promote osteoclastogenesis and recruit or reprogramme myeloid populations. In turn, TAMs and PMN-MDSCs can reinforce immunosuppression, angiogenesis and osteoclast activation through cytokine- and protease-mediated signalling. Osteoclasts therefore represent a central interface between tumour growth, immune remodelling and neural injury, rather than merely terminal effectors of bone destruction.

Osteoclast-mediated bone resorption is a major component of bone-to-nerve signalling. Resorption releases calcium and matrix-bound growth factors, while proton secretion produces a highly acidic local environment. Tumour-, stromal- and osteoclast-associated matrix metalloproteinases and other proteases further degrade the extracellular matrix and facilitate tumour invasion. These biochemical changes may activate acid-sensitive receptors on sensory nerve terminals, disrupt neural extracellular matrices and increase the susceptibility of adjacent nerve roots and spinal cord tissue to compression, inflammation and direct injury (Clézardin et al., 2021; Andriessen et al., 2021). Conversely, neural elements can actively modify the metastatic niche. Bone-metastatic tumours promote the sprouting of calcitonin gene-related peptide (CGRP)-positive sensory fibres, whereas sensory nerve-derived CGRP can stimulate tumour-cell proliferation through the CRLR/p38/HSP27 pathway (Park et al., 2024). The resulting reciprocal interaction establishes a pathological cycle in which tumour-induced bone destruction damages neural structures, while neural signals may further support tumour expansion.

EVs may both propagate and potentially interrupt this bone–nerve coupling cycle. Breast cancer-derived EVs enriched in miR-19a cooperate with integrin-binding sialoprotein to promote osteoclastogenesis and establish an osteolytic metastatic niche (Wu et al., 2021). Prostate cancer-derived small EVs similarly transfer miR-152–3p to osteoclast precursors, suppress MAFB and accelerate osteolytic bone destruction (Ma et al., 2021). EVs released by tumour-conditioned osteoclasts or myeloid cells may further transmit inflammatory and bone-remodelling signals among tumour cells, osteoblasts, osteoclasts and immune populations. By contrast, Schwann cell-derived EVs inhibited osteoclastogenesis through PTEN-dependent suppression of AKT signalling, and acidic-peptide surface modification increased their accumulation in bone (Yang et al., 2026). These observations identify EVs as candidate regulators of bidirectional nerve–bone communication and suggest three potential intervention points: inhibition of tumour-derived osteolytic EV signalling, reprogramming of the myeloid–osteoclast compartment and protection of adjacent neural tissue. However, these mechanisms have largely been demonstrated in bone-metastasis, peripheral nerve–bone or non-neoplastic bone-loss models. Their simultaneous antitumour, anti-osteolytic and neuroprotective effects remain to be validated in tumour-bearing orthotopic spinal models.

## 4 Extracellular vesicle-mediated repair of spinal tumor-associated neural injury: From mechanisms to interventions

**FIGURE 5:**
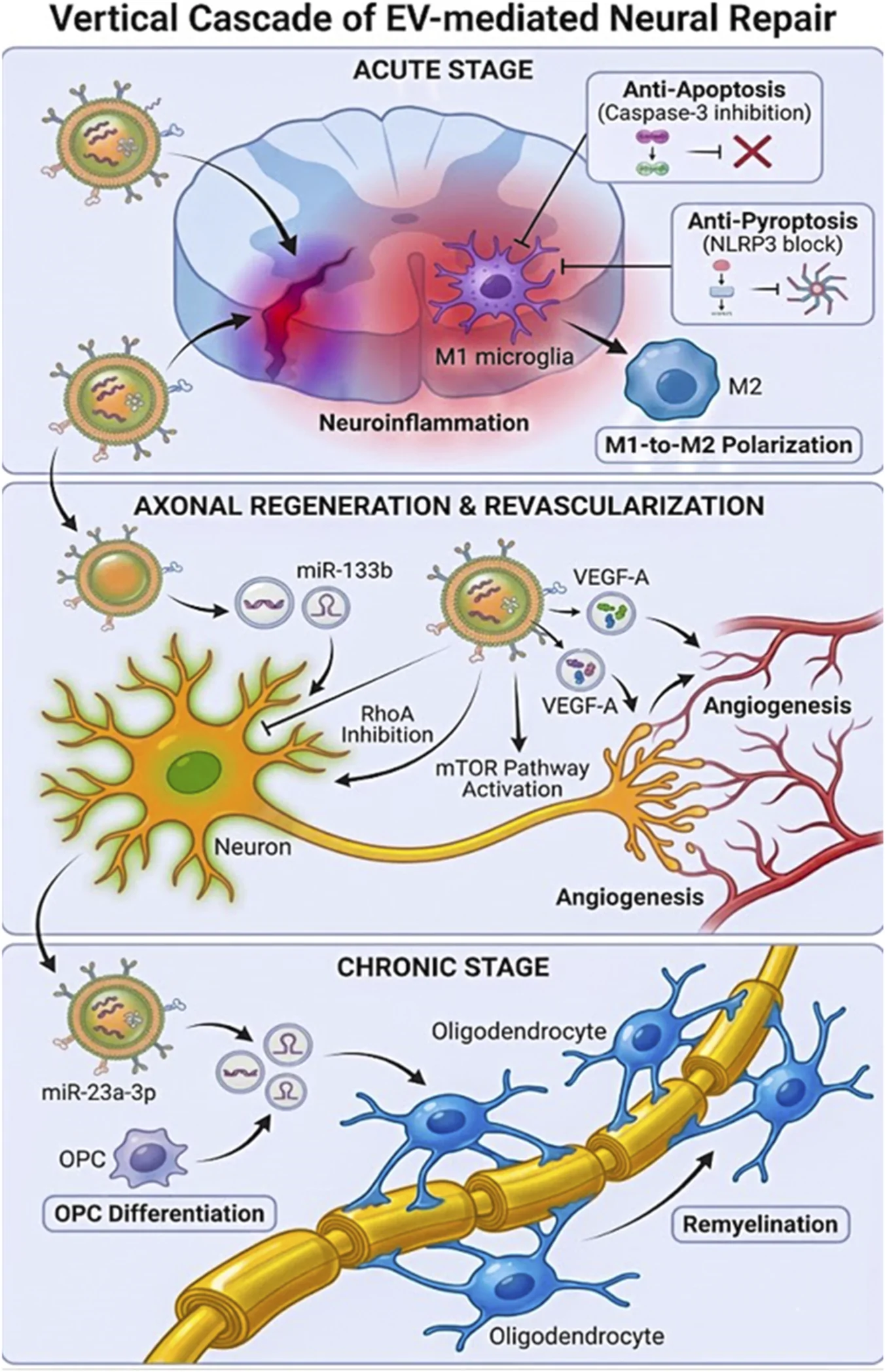
Vertical cascade of EV-mediated neural repair.

Neural repair after tumour resection involves a sequence of interdependent inflammatory, cell-death, axonal, vascular and myelin-remodelling processes. Figure 5 maps the EV-related evidence onto this temporal sequence. Direct evidence in *bona fide* spinal-tumor or post-resection MRD models remains scarce. Most studies summarized below use non-neoplastic SCI, chemical demyelination, peripheral nerve injury or central nervous system (CNS) tumor models. Within this evidence hierarchy, non-neoplastic neural-injury studies are classified as Level 4 unless otherwise specified. The evidence is interpreted by distinguishing mechanistic support from translational relevance, while also considering the risks of extrapolating these findings to a tumour-residual microenvironment. The mechanisms and evidence limitations of EV-mediated neural repair are summarized in Table 2.

**TABLE 2:** Mechanisms and evidence limitations of extracellular vesicles in repairing neural injury associated with spinal tumors.

Source (cell type)	Cargo	Repair mechanism and signalling pathway	Repair effect	Model context	Evidence level	Main limitation	References
Bone marrow MSCs	miR-125a	Targeted inhibition of IRF5 expression	Reduces neuronal apoptosis, alleviates inflammatory injury	Rat T10 spinal cord contusion (SCI)	Level 4	Lack of long-term follow-up data on chronic-phase efficacy and motor function recovery	(Chang et al., 2021)
Neural stem cells (NSCs)	miR-374–5p	Activates STK4 axis and induces autophagy	Inhibits neuronal apoptosis, improves motor scores	Rat/mouse SCI models	Level 4	Limited dose exploration; optimal therapeutic time window not yet defined	(Zhang and Han, 2022)
Adipose-derived stem cells (ADSCs)	miR-499a-5p	Targets JNK3/c-Jun pathway	Reduces apoptosis, maintains axonal integrity	Rat SCI model	Level 4	Primarily validated in the acute injury phase; lacking evidence of long-term neuroprotection	(Hwang et al., 2023)
Bone marrow MSCs	miR-497–5p	Inactivates TXNIP/NLRP3 inflammasome	Suppresses pyroptosis, necrosis and apoptosis	Rat T10 spinal cord contusion model	Level 4	Stability of vesicles in the complex and persistent chronic inflammatory microenvironment is questionable	(Xu et al., 2025a)
Bone marrow MSCs	miR-145–5p	Inhibits TLR4/NF-κB pathway activation	Reduces TNF-α/IL-6, attenuates inflammatory storm	Rat SCI inflammation model	Level 4	Lacks validation in a composite model of tumor-associated spinal cord injury (TA-SCI) following spinal tumor resection	(Jiang and Zhang, 2021)
M2 macrophages	miR-23a-3p (loaded with berberine)	Modulates PTEN/PI3K/AKT axis	Decreases pro-inflammatory factors, supports neuronal survival	Spinal cord contusion/sciatic nerve injury models	Level 4	Short half-life of free EVs in vivo limits long-acting anti-inflammatory capacity	(Gao et al., 2021)
Bone marrow MSCs	miR-133 b	Targeted inhibition of RhoA protein	Relieves regenerative inhibition and promotes axonal ingrowth	Rat T9 spinal hemisection/injury	Level 4	Relatively uniform animal model, difficult to fully mimic complex clinical compressive injury	(Li et al., 2018)
Engineered bone marrow MSCs	miR-26a	Inhibits PTEN, activates PI3K/AKT/mTOR	Enhances axonal regeneration and reduces glial scar	Mouse T9 spinal cord contusion model	Level 4	Insufficient in vivo pharmacokinetic data; barrier-crossing targeting efficiency still needs optimization	(Chen et al., 2021a)
ADSC/UCMSC	miR-199a-3p/145–5p	Activates NGF/TrkA signalling axis	Promotes neurite outgrowth and axonal bridging	Rat complete spinal transection/contusion models	Level 4	Difficult to breach the dense glial scar barrier to achieve deep neural parenchymal delivery	(Hwang et al., 2023)
iPSCs	let-7b-5p	Inhibits LRIG3/PI3K/Akt pathway	Reduces pyroptosis and promotes axonal extension	Mouse T10 spinal cord contusion model	Level 4	iPSC-derived vesicles face barriers in large-scale production and GMP-standardized preparation	(Liu J et al., 2024)
A2 astrocytes	miR-5121	Activates AKT2/mTOR/p70S6K	Promotes blood–spinal cord barrier (BSCB) repair and centripetal axonal growth	Traumatic SCI model	Level 4	Difficulty in primary glial culture expansion results in extremely low in vitro EV yield	(Wang et al., 2025)
Umbilical cord MSCs (hUCMSCs)	miR-23a-3p	Targets Tbr1/Wnt signalling pathway	Induces OPC differentiation and upregulates myelin proteins	Mouse LPC demyelination model	Level 4	Susceptible to reverse interference by EVs from activated microglia in the local microenvironment	(Qin et al., 2024)
Neural stem cells (NSCs)	VEGF-A	Direct binding to vascular endothelial receptors	Promotes angiogenesis and improves local blood supply	Rat/mouse SCI vascular models	Level 4	Lack of dynamic intervention data for different pathological stages (acute vs. chronic)	(Sintakova and Romanyuk, 2024)
BMSC + conductive hydrogel	Sustained release of EVs	Immunomodulation + physical scaffold guidance	Synergistically promotes axonal bridging and functional recovery	Complete spinal cord transection model	Level 4	Degradation rate of hydrogel scaffold is difficult to precisely match the timeline of endogenous neural regeneration	(Fan et al., 2022)

Figure 5 organizes EV-mediated neural repair as a temporally ordered cascade rather than as a set of isolated mechanisms. In the acute stage, EV cargoes may attenuate neuroinflammation and neuronal loss by shifting microglia and macrophages away from an M1-dominant phenotype and by modulating apoptosis and inflammasome-associated pyroptosis (Chang et al., 2021; Zhang and Han, 2022; Liang et al., 2022; Xu J. et al., 2025; Li et al., 2021). During the subsequent regenerative phase, EV-delivered miR-133 b and miR-26a can promote axonal growth through RhoA suppression and PTEN/AKT/mTOR signaling, whereas pro-angiogenic cargoes and immune-mediated vascular remodeling may support revascularization (Sintakova and Romanyuk, 2024; Li et al., 2018; Chen Y. et al., 2021; Wang et al., 2025; Fan et al., 2022). In the chronic stage, EV-mediated regulation of oligodendrocyte precursor-cell differentiation, including the miR-23a-3p/Tbr1/Wnt axis, may facilitate remyelination (Qin et al., 2024; Ji et al., 2024). These processes are biologically interdependent: early control of inflammation and cell death may establish a permissive environment for axonal and vascular repair, which in turn supports later circuit stabilization and myelin restoration. However, the cascade shown in Figure 5 is a mechanistic synthesis rather than a validated treatment sequence for spinal tumors. Most supporting studies used non-neoplastic spinal cord injury, demyelination, or *in vitro* models, and pro-survival, pro-angiogenic, or mTOR-activating EV cargoes could also protect residual tumor cells. Translation therefore requires phase-specific dosing, recipient-cell targeting, and concurrent assessment of neural recovery and oncological safety (Predominantly Level 4; *in vitro* components are outside Levels 1–5.)

**FIGURE 6:**
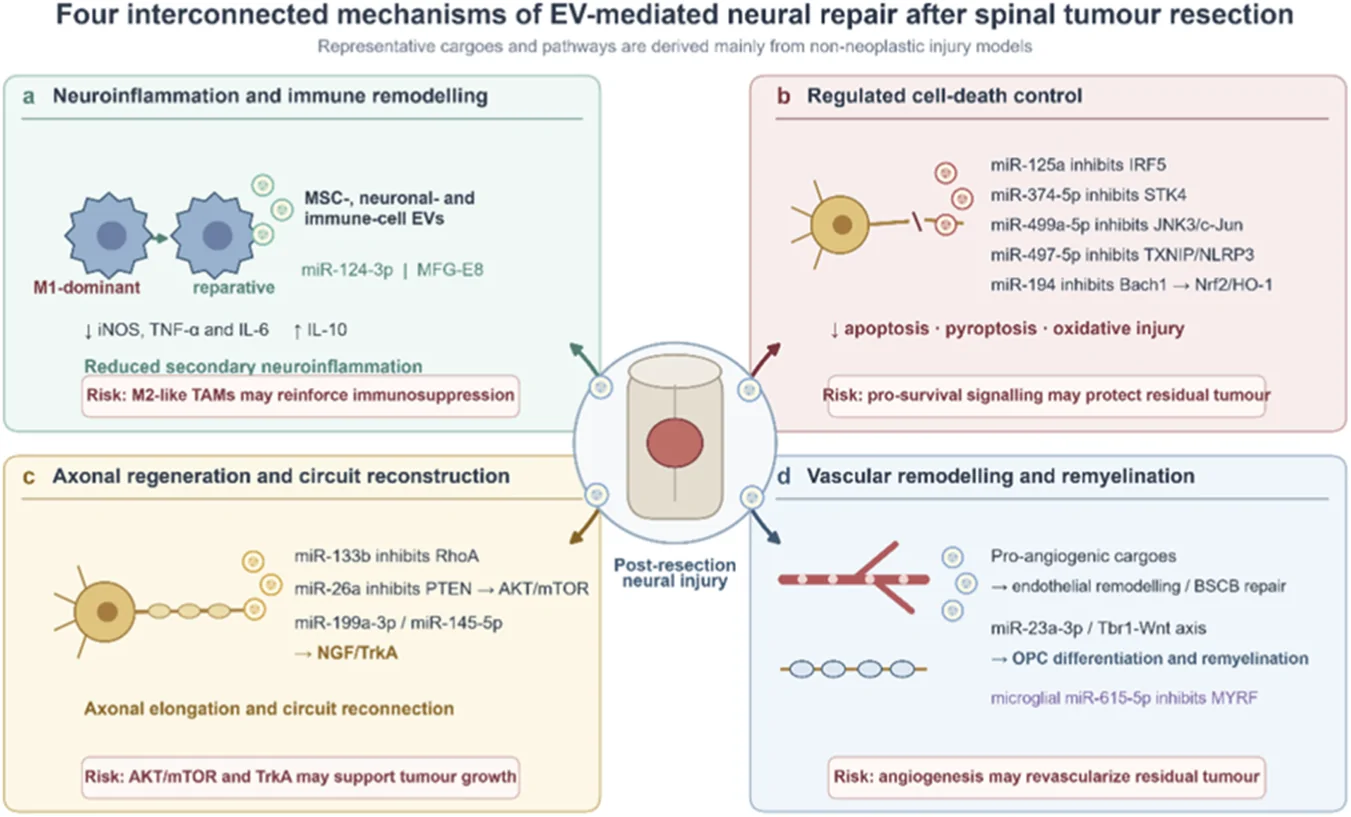
Four interconnected mechanisms of EV-mediated neural repair after spinal tumour resection. **(a)** Neuroinflammation and immune remodelling. **(b)** Regulated cell-death control. **(c)** Axonal regeneration and circuit reconstruction. **(d)** Vascular remodelling and remyelination. Solid coloured arrows indicate mechanistic links to postoperative neural injury, whereas red-bordered boxes indicate potential oncological risks. The schematic is a conceptual synthesis and does not represent a validated treatment sequence in spinal-tumour or minimal residual disease models.

To complement this temporal view, Figure 6 resolves EV-mediated neural repair into four interconnected mechanistic modules: neuroinflammation and immune-microenvironment modulation, regulation of neuronal and glial cell death, axonal regeneration and circuit reconstruction, and vascular remodelling coupled with remyelination. The figure links representative EV cargoes to recipient processes while distinguishing reparative mechanisms from potential residual-tumour risks (Sintakova and Romanyuk, 2024; Chang et al., 2021; Zhang and Han, 2022; Liang et al., 2022; Xu J. et al., 2025; Li et al., 2021; Li et al., 2018; Chen Y. et al., 2021; Wang et al., 2025; Fan et al., 2022; Qin et al., 2024; Ji et al., 2024) (Predominantly Level 4; contextual reviews and *in vitro* components are outside Levels 1–5.)

The four mechanisms are mechanistically linked rather than biologically isolated. Early modulation of inflammation and cell death may influence the conditions required for subsequent axonal, vascular and myelin repair. However, pro-survival, immunomodulatory and pro-angiogenic signals may also favour the persistence of residual tumour cells. Interpretation of these mechanisms requires simultaneous consideration of EV source, representative cargo, recipient cell, experimental model and the corresponding tumour-related safety boundary.

### 4.1 Reversing pro-tumorigenic neuroinflammation and modulating the immune microenvironment

The postoperative setting includes acute inflammation, ischemic injury and neuronal death after mechanical decompression, but it may also contain residual tumor cells, tumor-associated macrophages (TAMs) and local immunosuppressive networks. EV-mediated neural repair therefore cannot be equated with regeneration observed in conventional SCI models. Potential neurological benefits must be weighed against the possibility of supporting residual-tumor survival or recurrence. The following discussion retains a target-oriented framework based on the pathophysiological sequence of neural repair while emphasizing differences in evidence quality across experimental models.

Tumor-derived EVs: drivers of pro-tumor inflammation and immune escape. EVs released by tumor cells can create a local microenvironment that is both inflammatory and immunosuppressive. In orthotopic intracranial GL261 glioma models and U87-MG glioma cell-microglia co-cultures, tumor-derived EVs contain miRNAs such as miR-21 and miR-451. After uptake by resident microglia and infiltrating monocytes or macrophages, these miRNAs promote proliferation and a tumor-associated immunosuppressive phenotype. This phenotype is characterized by reduced TNF-α and IL-6 and increased immunosuppressive mediators. In high-grade glioma, the same process may also promote epithelial-mesenchymal transition (EMT), oxidative stress and disruption of the blood-brain or blood-spinal cord barrier (van der Vos et al., 2016) (Level 3 for the intracranial tumour model; *in vitro* co-culture evidence is outside Levels 1–5). These findings suggest that tumor-derived EVs can actively contribute to neuroinflammation and immune escape rather than merely reflect tumor progression.

In GL261 tumor-bearing mice, tumor-derived EVs suppress innate and adaptive antitumor immunity by inhibiting T-cell activation, promoting M2-like macrophage polarization and inducing immune-cell apoptosis (Hellwinkel et al., 2016; Abels et al., 2019) (Level 3). Cerebrospinal-fluid tumor EVs and cargoes such as EGFRvIII may also serve as malignancy markers and contribute to persistent postoperative neuroinflammation (Figueroa et al., 2017) (Level 1 human diagnostic evidence; not therapeutic evidence). However, these findings come mainly from intracranial glioma rather than orthotopic spinal tumors or spinal metastases. Delivery of miR-21/451 may vary between individuals, and EGFRvIII expression in breast- or lung-derived spinal metastases may differ from that in glioma. The evidence therefore supports a mechanistic role in neuro-oncological inflammation but does not establish the same mechanism after spinal-tumor surgery.

Stem cell-, neuron-, and immune cell-derived EVs: regulators of the microenvironment. In contrast to tumor-derived EVs, which may promote immune escape and chronic inflammation, EVs derived from stem cells, M2 macrophages, and neurons have shown anti-inflammatory, immunomodulatory, and pro-regenerative effects in several neural injury models. These EVs may counteract the harmful effects of tumor-derived EVs by promoting resolution of inflammation, regulating microglial or macrophage polarization, and improving the local regenerative niche. Nevertheless, most of these protective effects have been demonstrated in simple SCI, peripheral nerve injury, or *in vitro* inflammatory models, and direct validation in spinal tumor resection-associated neural injury is still lacking.

Regulatory effects of stem cell-derived EVs. In a rat T9-T10 spinal cord contusion model, bone marrow mesenchymal stem cell-derived EVs (BMSC-EVs) suppressed A1 neurotoxic reactive astrocytes, reduced nitric oxide release from microglia, and regulated microglial polarization through the NF-κB pathway, with upregulation of Arg-1/IL-10 and downregulation of iNOS/TNF-α/IL-6, resulting in reduced glial scar formation (Wang et al., 2018) (Level 4). In mouse spinal cord contusion and LPS-stimulated BV-2 cell models, umbilical cord MSC-derived EVs similarly inhibited phosphorylation of NF-κB and MAPK pathway components, including p65, p38, JNK, and ERK, thereby reducing inflammatory mediator release (Luan et al., 2024) (Level 4 for the contusion model; *in vitro* BV-2 evidence is outside Levels 1–5). In addition, miR-145–5p delivered by BMSC-EVs specifically targeted TLR4 and inhibited activation of the TLR4/NF-κB pathway, significantly reducing inflammation in rat SCI models (Jiang and Zhang, 2021) (Level 4). These studies support the anti-inflammatory and anti-scarring potential of MSC-EVs in non-neoplastic SCI. However, SCI models lack residual tumor cells, TAMs, and tumor-associated immunosuppressive networks, so their safety and efficacy cannot be directly assumed in the postoperative spinal tumor context.

Neuron-derived EVs may participate in immune surveillance in the setting of spinal tumor resection accompanied by spinal cord injury and inflammation. *In vitro* studies have shown that physiological neuron-derived EVs (NDEVs) can help maintain basal immune tolerance in the central nervous system by downregulating pro-inflammatory mediators such as iNOS, TNF-α, and IL-6, while selectively upregulating the anti-inflammatory cytokine IL-10 (Peng et al., 2021) (outside Levels 1–5; *in vitro* evidence). After trauma, NDEVs may activate an endogenous protective axis by delivering enriched miR-124–3p, which targets and degrades myosin heavy chain 9 (MYH9) in microglia. This blocks phosphorylation of the PI3K/AKT/NF-κB pathway and suppresses classical M1 microglial activation and A1 reactive astrocyte proliferation (Jiang et al., 2020) (Level 4). However, the effects of NDEVs are not always protective. In tumor or severe inflammatory microenvironments, neuron-derived EVs may undergo pathological changes. For example, highly stressed neurons may release pathological vesicles enriched in miR-21–5p. After uptake by microglia, these abnormal EVs can drive microglia toward a pathogenic M1 pro-inflammatory phenotype, thereby aggravating neuroinflammation and secondary injury (Yin et al., 2020) (outside Levels 1–5; *in vitro* evidence). Moreover, when these miR-21-5p-enriched vesicles are taken up by neural stem cells (NSCs) or glial precursor cells around the lesion, they may suppress endogenous SMAD7 expression and aberrantly amplify TGF-β/Smad signaling. This process may not only impair oligodendrocyte maturation and normal myelin remodeling, but also induce abnormal astrocytic differentiation of precursor cells, ultimately leading to excessive dense glial scar formation (Han et al., 2023) (Level 4). Therefore, the role of NDEVs in neural repair is highly context-dependent and should not be regarded as uniformly protective.

Regulatory effects of immune cell-derived EVs. Macrophage reprogramming is an important mechanism of EV-mediated neuroinflammatory control. M2 macrophage-derived EVs have anti-inflammatory activity and can carry drugs or miRNAs. In spinal-cord contusion and sciatic-nerve injury models, berberine-loaded M2-EVs reduced TNF-α and IL-1β through the miR-23a-3p/PTEN/PI3K/AKT axis (Gao et al., 2021) (Level 4). Schwann cell-derived EV MFG-E8 can also promote an M2-like anti-inflammatory phenotype. In subcutaneous colon cancer and breast-cancer lung-metastasis models, selected immune-cell EVs reprogrammed TAMs toward an antitumor state (Zhang et al., 2024) (Level 5 for the subcutaneous colon-cancer model; Level 3 for the breast-cancer lung-metastasis model) (Zhao et al., 2022) (Level 5). CRISPR-modified stem-cell EVs have also been designed to neutralize TNF-α and reduce local inflammatory toxicity (Wang et al., 2022) (Level 4). These findings require caution because the evidence comes mainly from mechanical SCI or non-spinal tumor models. In TA-SCI, reparative M2 polarization may instead converge on a pro-tumor TAM phenotype. Additional barriers include transient TAM reprogramming (<72 h), BSCB restriction, off-target CRISPR editing and the low *in vitro* yield of neuron-derived EVs (<10^8^ particles/10^6^ cells).

### 4.2 Inhibition of apoptosis and pyroptosis: Acute-phase neuroprotection

In the acute phase after spinal tumor resection, neurological preservation requires control of apoptosis, necroptosis, pyroptosis, and ferroptosis triggered by compression, ischemia-reperfusion, and inflammation. In non-neoplastic SCI models, BMSC-derived EVs carrying miR-125a, NSC-derived EVs carrying miR-374–5p, hypoxia-preconditioned ADSC-derived EVs enriched in miR-499a-5p, and BMSC-derived EVs carrying miR-497–5p have modulated IRF5, STK4, JNK3/c-Jun, and TXNIP/NLRP3 signaling, respectively (Chang et al., 2021; Zhang and Han, 2022; Liang et al., 2022; Xu J. et al., 2025) (Level 4). In an *in vitro* oxygen-glucose deprivation/reoxygenation model, MSC-derived exosomal miR-194 additionally suppressed Bach1 and activated Nrf2/HO-1 signaling (Li et al., 2021) (outside Levels 1–5; *in vitro* oxygen-glucose deprivation/reoxygenation model). These studies support acute neuroprotective mechanisms across SCI and ischemia-reperfusion settings, but they do not establish efficacy or oncological safety after spinal-tumor surgery. When residual tumor cells may be present, broad inhibition of cell death or activation of survival signaling could also impair tumor-cell clearance and local immune surveillance.

Anti-apoptotic and anti-necrotic effects. In SD rat models of T10 spinal cord contusion, miR-125a enriched in BMSC-EVs exerted neuroprotective effects by targeting and downregulating IRF5 (Chang et al., 2021) (Level 4). EVs derived from neural stem cells (NSCs) delivered miR-374–5p and inhibited neuronal apoptosis through the STK4 axis (Zhang and Han, 2022) (Level 4). EVs from adipose-derived stem cells (ADSCs) reduced apoptosis by delivering miR-499a-5p, which targets the JNK3/c-Jun pathway (Hwang et al., 2023) (contextual review; outside Levels 1–5). For necrosis and pyroptosis, BMSC-EVs carrying miR-497–5p have been reported to inhibit apoptosis and necrotic progression in neural cells at the lesion site by inactivating the TXNIP/NLRP3 inflammasome pathway (Xu J. et al., 2025) (Level 4). These studies support the ability of EVs to regulate acute neural cell death at the mechanistic level, but they also have clear limitations. Most studies used single-dose administration, and the therapeutic window, dose-response relationship, and safety of repeated dosing remain insufficiently defined. The durability of therapeutic effects in the chronic phase, particularly beyond 8 weeks, also lacks systematic evidence (Chang et al., 2021). Therefore, these findings are better interpreted as mechanistic support for acute neuroprotection rather than direct evidence of translational feasibility after spinal tumor surgery.

Inhibition of pyroptosis and ferroptosis. In mouse T10 contusion and microglial pyroptosis models, let-7b-5p-enriched induced-pluripotent-stem-cell EVs inhibited the LRIG3/PI3K/Akt pathway and reduced pyroptosis (Liu J. et al., 2024) (Level 4). In ischemia-reperfusion injury, EV-delivered miR-194 targeted Bach1, relieved suppression of Nrf2/HO-1 signaling and reduced oxidative and ferroptosis-related injury (Li et al., 2021) (outside Levels 1–5; *in vitro* ischemia-reperfusion model). These observations support acute neuroprotection through inflammasome and oxidative-stress pathways. However, the anti-ferroptotic evidence derives mainly from cerebral ischemia models, including middle cerebral artery occlusion (MCAO). These models do not reproduce the combination of decompression injury, postoperative inflammation and residual tumor-associated immunity. Translation therefore requires tumor-bearing spinal models that assess neurological recovery, residual-tumor growth, immune surveillance and long-term safety in parallel.

### 4.3 Driving axonal regeneration and circuit reconstruction

In the subacute to chronic phases of injury, stimulating severed or damaged neurons to extend new axons (neurite outgrowth) across the lesion core is central to functional recovery. Extracellular vesicles (EVs) can directly activate or disinhibit key intracellular regenerative signaling cascades.

In a rat T9 spinal hemisection model, EV-delivered miR-133 b promotes axonal regeneration by suppressing RhoA, thereby activating downstream ERK1/2, STAT3, and CREB pathways (Li et al., 2018) (Level 4). Similarly, in a murine T9 contusion model, EV-overexpressed miR-26a inhibits PTEN, subsequently activating the PI3K/AKT/mTOR pathway to enhance axonal and neural regeneration while attenuating glial scarring (Chen Y. et al., 2021) (Level 4). These strategies do not carry equivalent oncological risks. PTEN suppression with PI3K/AKT/mTOR activation should be regarded as a high-risk regenerative mechanism when minimal residual disease is possible, because this axis is an established driver of proliferation, survival, therapeutic resistance, and metastatic progression in breast, lung, and prostate cancers (Khorasani et al., 2024; Sanaei et al., 2022; Cham et al., 2021) (contextual cancer-pathway reviews; outside Levels 1–5). By contrast, miR-133 b suppresses RhoA. Although RhoA activity can support tumor-cell invasion, experimental inhibition of RhoA has reduced aggressive behavior in breast, lung, and prostate cancer models (Tsubaki et al., 2021; Liu et al., 2017; Hodge et al., 2003) (Level 3 where based on non-spinal tumour models). The risk of RhoA-suppressive EVs is therefore context-dependent rather than intrinsically tumor-promoting: a tumor-cell effect may be favorable, whereas consequences in fibroblasts, endothelial cells, immune cells, and neural cells, as well as the recipient-specific activation of ERK1/2–STAT3–CREB, remain unresolved. Accordingly, PTEN/mTOR-activating EVs require stringent recipient-cell restriction and exclusion of residual tumor, whereas RhoA-suppressive EVs require compartment-specific validation rather than automatic classification as high risk.

Furthermore, miR-199a-3p and miR-145–5p within ADSC- and UCMSC-derived EVs can directly activate the NGF/TrkA signaling axis to stimulate neurite outgrowth (Hwang et al., 2023) (contextual review; outside Levels 1–5). This pathway warrants an intermediate, tumor-type-specific risk classification rather than an assumption of safety. NGF/TrkA signaling has mitogenic, anti-apoptotic, angiogenic, and invasion-associated effects in breast cancer (Bruno et al., 2022) (contextual review; outside Levels 1–5) and promotes proliferation, invasiveness, and epithelial–mesenchymal transition in castration-resistant prostate cancer models (Di Donato et al., 2019) (Level 3 non-spinal prostate-cancer model). In lung cancer, NGF and TrkA expression is histology-dependent, with the strongest association reported in squamous cell carcinoma, while direct functional and clinical evidence remains limited (Gao et al., 2018) (non-spinal lung-cancer evidence; model-specific level not established). Thus, oncogenic concern is supported in breast and advanced prostate cancer but is less conclusive and subtype-dependent in lung cancer, and none of these findings has been validated in spinal metastasis models. Among astrocyte-derived EVs, A2-type EVs harboring miR-5121 similarly activate the AKT2/mTOR/p70S6K pathway to facilitate blood–spinal cord barrier repair and axonal growth (Wang et al., 2025) (Level 4). Because this mechanism converges on mTOR, it should remain in the high-risk category despite the astrocytic source unless uptake and pathway activation can be confined to neural or endothelial recipient cells. The safety of introducing either cargo into a postoperative tumor-resection cavity therefore requires simultaneous assessment of neural repair and residual-tumor growth in dual-pathology models.

To overcome the immense spatial barriers at the lesion site, engineering strategies provide essential adjunctive support. For instance, encapsulating BMSC-EVs into a conductive hydrogel to form an EV-hydrogel composite system supports axonal bridging in complete spinal cord transection models, enhancing the local delivery and retention of EVs. From a practical clinical perspective, however, a biomechanical mismatch persists between these hydrogel composites and the highly dynamic anatomical environment of the authentic spinal canal. How to ensure that biomaterial scaffolds effectively bridge severed neural stumps without inadvertently providing a physical niche for the colonization of residual cancer cells represents the paramount challenge for future bioengineering designs.

### 4.4 Promoting angiogenesis and remyelination

Long-term functional recovery of neural tissue is fundamentally dependent upon the reconstruction of blood supply and the repair of insulating myelin sheaths. This endeavor demands not only the regeneration of parenchymal cells but also the synergistic orchestration of the vascular and glial compartments.

The EV-mediated remodeling of the immune microenvironment (e.g., inducing M2 macrophage polarization) can indirectly foster angiogenesis, reportedly increasing vascular density by up to 60% (Fan et al., 2022) (Level 4). Furthermore, neural stem cell (NSC)-derived EVs innately harbor VEGF-A, enabling them to directly stimulate angiogenesis and emulate the vascular repair processes requisite following tumor resection (Sintakova and Romanyuk, 2024) (contextual review; outside Levels 1–5). In a postoperative minimal-residual-disease setting, however, these effects cannot be treated as uniformly reparative. VEGF-driven neovascularization and sustained M2-like polarization may support tumor perfusion and immune escape, while the direction of EV-mediated angiogenesis also varies by vesicle source, as illustrated by antiangiogenic menstrual-stem-cell EVs in a prostate-tumor model (Alcayaga-Miranda et al., 2016) (Level 5). Unrestricted proangiogenic or M2-polarizing EV administration should therefore be classified as high risk when residual breast, lung, or prostate cancer is suspected and should be restricted to local, time-limited delivery after oncological control has been demonstrated.

Regarding myelin repair, in murine lysolecithin (LPC)-induced demyelination models and *in vitro* oligodendrocyte precursor cell (OPC) differentiation systems, EVs derived from human umbilical cord MSCs deliver miR-23a-3p to target Tbr1 and Wnt signaling. This mechanism promotes the differentiation of OPCs into mature oligodendrocytes and upregulates the expression of myelin-associated proteins, thereby facilitating robust remyelination (Qin et al., 2024) (Level 4 for the LPC demyelination model; *in vitro* OPC evidence is outside Levels 1–5). However, it is crucial to recognize that this regenerative process can be severely impeded within a complex inflammatory microenvironment. Specifically, EVs secreted by activated microglia may contain miR-615–5p, a molecule that targets the myelin regulatory factor MYRF. This molecular interference obstructs oligodendrocyte differentiation, ultimately exerting a profound inhibitory effect on remyelination (Ji et al., 2024) (Level 4 where based on non-neoplastic neural-injury models). It must be explicitly noted that current evidence regarding EV-mediated remyelination and angiogenesis is entirely extrapolated from non-neoplastic SCI or chemical demyelination (e.g., LPC-induced) models. Interpreting these findings within a spinal tumor context remains highly speculative. In fact, robust induction of angiogenesis—while beneficial for neural repair—poses a perilous risk of simultaneously vascularizing residual micro-tumors.

## 5 Representative paradigms and emerging trends over the past five years

Recent EV research in spinal tumors and associated neural injury has expanded from isolated mechanistic studies toward combined targeting, delivery and regenerative strategies.

### 5.1 Precision targeting and payload innovation

Recent work has shifted from non-specific cargo loading toward surface engineering intended to improve lesion-directed delivery across the vertebral matrix and BSCB. Four approaches are particularly relevant: donor-cell genetic display, post-isolation chemical conjugation, lipid insertion and non-covalent modification. Genetic display can provide stable and oriented membrane presentation, but it depends on donor-cell engineering and EV biogenesis, which may introduce clone- and batch-dependent variation. Covalent conjugation provides durable, modular attachment, but non-selective reactions may alter membrane proteins, lipids or permeability and require removal of residual reagents. Lipid insertion is rapid and generally less disruptive, although high amphiphile loading may alter membrane packing and inserted ligands may desorb in serum. Non-covalent methods are operationally simple, but surface occupancy may be heterogeneous and binding may weaken under physiological shear or ionic conditions (Liang et al., 2021; Zhang et al., 2023; Di Ianni et al., 2025).

These trade-offs require method-specific quantitative assessment. Coupling or display efficiency should be reported as both the proportion of ligand-positive EVs and, where possible, the number of ligand molecules per vesicle. It should not be conflated with biological targeting efficiency, which is measured by receptor-dependent uptake or lesion-to-organ accumulation. Manufacturing assessment should additionally report particle recovery, size distribution, morphology, membrane permeability, EV-marker and cargo retention, ligand stability, batch-to-batch variation and potency after scale-up (Welsh et al., 2024; Di Ianni et al., 2025; Xu G. et al., 2025; Carney et al., 2025; Goldbloom-Helzner et al., 2024a).

In the Angiopep-2 (Ang2) system, the targeting peptide was introduced by biologically engineering M2 microglia, allowing Ang2 to be displayed on released EVs and supporting LRP1-mediated accumulation at spinal cord injury sites (Kong et al., 2025). This represents donor-cell genetic display rather than post-isolation chemical conjugation. Although the study demonstrated preferential lesion localization and retained standard EV characteristics, it did not report ligand molecules per EV, the proportion of Ang2-positive EVs, batch-to-batch variability or scaled production yield. The imaging data therefore establish relative targeting performance, but not quantitative coupling efficiency. Moreover, the model involved non-neoplastic spinal cord injury; targeting and efficacy in tumour-bearing spinal tissue remain unproven.

HER2 dual-targeted EVs were generated from stable HEK-293 producer cells co-expressing miR-HER2-E1 and a C1C2-anchored HER2-binding peptide, thereby combining genetic surface display with endogenous miRNA loading (Wang et al., 2020). HER2 binding was confirmed by ELISA, uptake was enhanced in HER2-positive cells, and 3 μg and 30 μg per mouse produced similar tumour suppression in subcutaneous SK-OV-3 xenografts, suggesting a plateau in biological efficacy. However, the study did not quantify peptide copies per EV or the percentage of ligand-positive EVs, and formal batch comparability and scale-up were not reported. Preserved particle-size distribution and EV markers support product identity, but do not by themselves establish membrane integrity throughout manufacturing or reproducibility at GMP scale.

A complementary lipid-insertion strategy used macrophage-derived EV membranes to coat doxorubicin-loaded PLGA nanoparticles and inserted a c-Met-binding peptide through DSPE-PEG2000 (Li et al., 2020). In MDA-MB-231 cells, EV coating increased intracellular doxorubicin accumulation by 3.31-fold relative to PLGA nanoparticles, and c-Met functionalization produced a further approximately twofold increase relative to the non-targeted hybrid. The apoptotic fraction increased from 29.27% to 39.73%, and *in vivo* tumour accumulation was 1.62-fold higher than that of the non-targeted hybrid and 2.22-fold higher than that of PLGA nanoparticles. These results quantify targeting performance, not peptide-insertion efficiency. Ligand occupancy, post-insertion stability, batch variability and scale-up recovery were not reported, and the orthotopic mammary model provides Level 3 rather than spinal-tumour evidence.

PSMA-targeted exosome mimetics were produced by nucleofection or lentiviral engineering of U937 cells to display the anti-PSMA peptide WQPDTAHHWATL, followed by serial extrusion and size-exclusion chromatography (Severic et al., 2021). Targeted and control mimetics had comparable hydrodynamic diameters (170–180 nm), polydispersity indices below 0.2 and zeta potentials of approximately −18 mV. Nanoparticle-tracking analysis showed mean diameters of 133–135 nm and concentrations of 2.16–2.49 × 10^10^ particles/mL. Spherical morphology and retention of LFA-1, Moesin, TSG101 and CD63 supported membrane and protein integrity after extrusion. Uptake was enhanced in PSMA-positive LNCaP and C4-2 B cells, and targeted mimetics showed greater accumulation in subcutaneous C4-2 B tumours than non-targeted mimetics. However, peptide density, the magnitude of the accumulation gain and batch variation were not reported. Extrusion may improve yield, but producer-cell control, repeated mechanical processing and chromatographic purification remain potential sources of variability. The evidence derives from subcutaneous C4-2 B tumors rather than bone metastasis or orthotopic spinal disease.

Collectively, the HER2, PSMA and Ang2 studies demonstrate receptor-dependent uptake or lesion enrichment, but none provides a standardized, directly comparable coupling-efficiency metric. Their translational relevance is further constrained by the absence of vertebral bone-metastasis or orthotopic spinal-tumour validation. Future studies should therefore combine single-vesicle ligand quantification (Goldbloom-Helzner et al., 2024b) with predefined release criteria for membrane integrity, cargo retention, batch comparability, scale-dependent yield and *in vivo* targeting. A comparative summary of the major EV surface-engineering strategies, including coupling or display efficiency, membrane integrity, reproducibility, scalability and model relevance, is provided in Table 3.

**TABLE 3:** Comparative evaluation of major EV surface-engineering strategies relevant to spinal tumour therapy.

Strategy and representative system	Coupling/display efficiency and quantitative targeting	Membrane integrity	Reproducibility and scalability	Model relevance and principal limitation	Source
Donor-cell genetic fusion: C1C2-anchored HER2-binding peptide on HEK-293 EV s (Wang et al., 2020)	Surface display was confirmed by immunofluorescence and Western blotting, but standardized ligand density, percentage of modified EVs and batch-to-batch display efficiency were not reported	Parent EV morphology and size distribution were retained after genetic modification and drug loading, supporting preservation of vesicle identity; however, formal membrane-integrity assays were limited	This strategy is genetically programmable and potentially reproducible after establishment of a stable donor-cell system, but scale-up depends on cell-line stability, EV yield and release consistency	HER2-positive breast-cancer models. Useful proof-of-concept for receptor-directed EV targeting, but not yet validated in spinal tumour models or under bone-metastatic pharmacokinetic constraints	(Wang et al., 2020)
Donor-cell biological display: Angiopep-2-modified M2-microglial EVs (Kong et al., 2025)	Targeting performance was supported by enhanced blood-brain-barrier penetration and glioma accumulation, but ligand copy number and the proportion of Angiopep-2-positive EVs were not quantified	EV morphology and marker expression were preserved after donor-cell engineering; the approach avoids harsh post-isolation chemistry and is therefore relatively membrane-sparing	Requires stable generation of engineered donor cells and controlled M2-microglial phenotype. Scalability is feasible in principle but may be affected by donor-cell state and culture conditions	Glioblastoma orthotopic models. Relevant to neuroaxis delivery, but extrapolation to spinal tumours requires validation across the blood-spinal-cord barrier and spinal tumour microenvironment	(Kong et al., 2025)
Genetic display plus serial extrusion: anti-PSMA peptide WQPDTAHHWATL on U937-derived exosome mimetics (Severic et al., 2021)	PSMA targeting was demonstrated by receptor-selective uptake and antitumour activity, but coupling efficiency was inferred from engineered membrane display rather than reported as ligand copies per vesicle or modified-vesicle fraction	Serial extrusion increases vesicle yield but can partly remodel the membrane compared with native EVs; therefore, membrane integrity should be verified by size, morphology, protein-marker retention and leakage assays	Extrusion improves production yield and may support scale-up, but mechanical processing introduces variability in vesicle size, cargo retention and surface-protein preservation	PSMA-positive prostate-cancer models. Relevant to targeted EV-mimetic delivery, but PSMA expression heterogeneity and absence of spinal tumour validation remain important limitations	(Severic et al., 2021)
Post-isolation covalent conjugation: LJM-3064 aptamer via sNHS-DBCO amide/click chemistry (Goldbloom-Helzner et al., 2024b)	Conjugation efficiency was quantified: ligand-positive EVs reached approximately 43% by ExoView and 30%–33% by EV flow cytometry under optimized amide-reaction conditions	Post-isolation chemistry enables controlled ligand attachment but can affect membrane proteins or vesicle surface charge; integrity should be assessed using morphology, particle size, marker retention and cargo leakage	Chemistry-based modification is modular and analytically traceable. Reproducibility depends on reaction stoichiometry, residual reagent removal and standardized EV input normalization	Methodological EV surface-engineering benchmark. Strong for comparing conjugation chemistry, but therapeutic performance in spinal tumour models remains untested	(Goldbloom-Helzner et al., 2024b)
Lipid insertion: DSPE-PEG2000-c-Met peptide on macrophage-EV-coated PLGA nanoparticles (Li et al., 2020); LJM-3064/DSPE-PEG benchmark (Goldbloom-Helzner et al., 2024b)	c-Met peptide insertion improved cellular uptake and tumour accumulation; LJM-3064/DSPE insertion reached up to 45% ligand-positive EVs by ExoView and 61% by EV flow cytometry	Lipid insertion is relatively mild and preserves the EV membrane more readily than covalent chemistry, but excessive ligand-lipid insertion may alter membrane fluidity or protein corona formation	This method is simple and scalable because ligand-lipid conjugates can be mixed with EV membranes after isolation. Reproducibility depends on ligand-lipid purity, insertion ratio and EV concentration	c-Met-positive triple-negative breast-cancer models and surface-engineering benchmarks. Useful for scalable targeting, but spinal tumour penetration and long-term biodistribution need confirmation	(Li et al., 2020; Goldbloom-Helzner et al., 2024b)
Non-covalent modification: electrostatic adsorption, affinity adaptors or receptor-ligand assembly (Zhang et al., 2023; Di Ianni et al., 2025; Goldbloom-Helzner et al., 2024a)	Coupling efficiency is usually formulation-dependent and less stable than covalent or lipid-insertion approaches; quantitative reporting should include bound-ligand fraction, ligand retention in serum and uptake selectivity.	Non-covalent methods are generally membrane-sparing because they avoid chemical reactions, but ligand shedding and surface-charge changes may affect EV identity and biodistribution	Technically simple and adaptable, but batch reproducibility can be weaker because ligand loading depends on buffer, pH, ionic strength, EV surface charge and incubation conditions	Mainly platform-level and disease-model studies. Useful when preserving vesicle membranes is prioritized, but weaker ligand stability may limit translational use unless retention is rigorously validated.	(Zhang et al., 2023; Di Ianni et al., 2025; Goldbloom-Helzner et al., 2024a)

### 5.2 Multifunctional composite delivery systems

EV-integrated hydrogels may address the short *in vivo* half-life of free vesicles. Conductive or adhesive scaffolds can provide local retention and structural support while EV cargoes regulate microglial polarization, axonal repair and microvascular remodeling (Fan et al., 2022).

Plant-derived EV-like nanoparticles have also been incorporated into hydrogels. In SCI models, Sophora japonica-derived nanovesicles reduced local reactive oxygen species (ROS) and were associated with improved motor-tract recovery (Chen J. et al., 2023) (Level 4). Their safety and efficacy in tumor-bearing spinal models remain unknown.

### 5.3 Pioneering clinical translation

Engineered EVs carrying KRASG12D-targeting siRNA have entered first-in-human evaluation (Kalluri et al., 2025). MSC-derived EVs are also being investigated for neural repair. In facial-nerve injury models, MSC-EVs promoted axonal regeneration and early functional recovery while modulating local macrophage polarization (Xue et al., 2024) (Level 4 related peripheral-nerve injury model). These peripheral-nerve findings do not establish efficacy after spinal-tumor surgery.

Early exploratory studies have evaluated localized intraparenchymal administration of placental MSC-derived EVs in patients with acute ischemic stroke after decompressive craniectomy (Dehghani et al., 2022) (Level 1 human clinical evidence; CNS injury rather than spinal-tumour therapy). These data provide preliminary safety information for EV delivery in CNS injury but do not demonstrate efficacy in spinal tumors. Overall, the field is moving toward multidimensional interventions, yet spatiotemporal separation of antitumor and regenerative effects and reproducible GMP-grade manufacturing remain unresolved.

## 6 Safety and biological risk considerations of extracellular vesicle therapeutics

**FIGURE 7:**
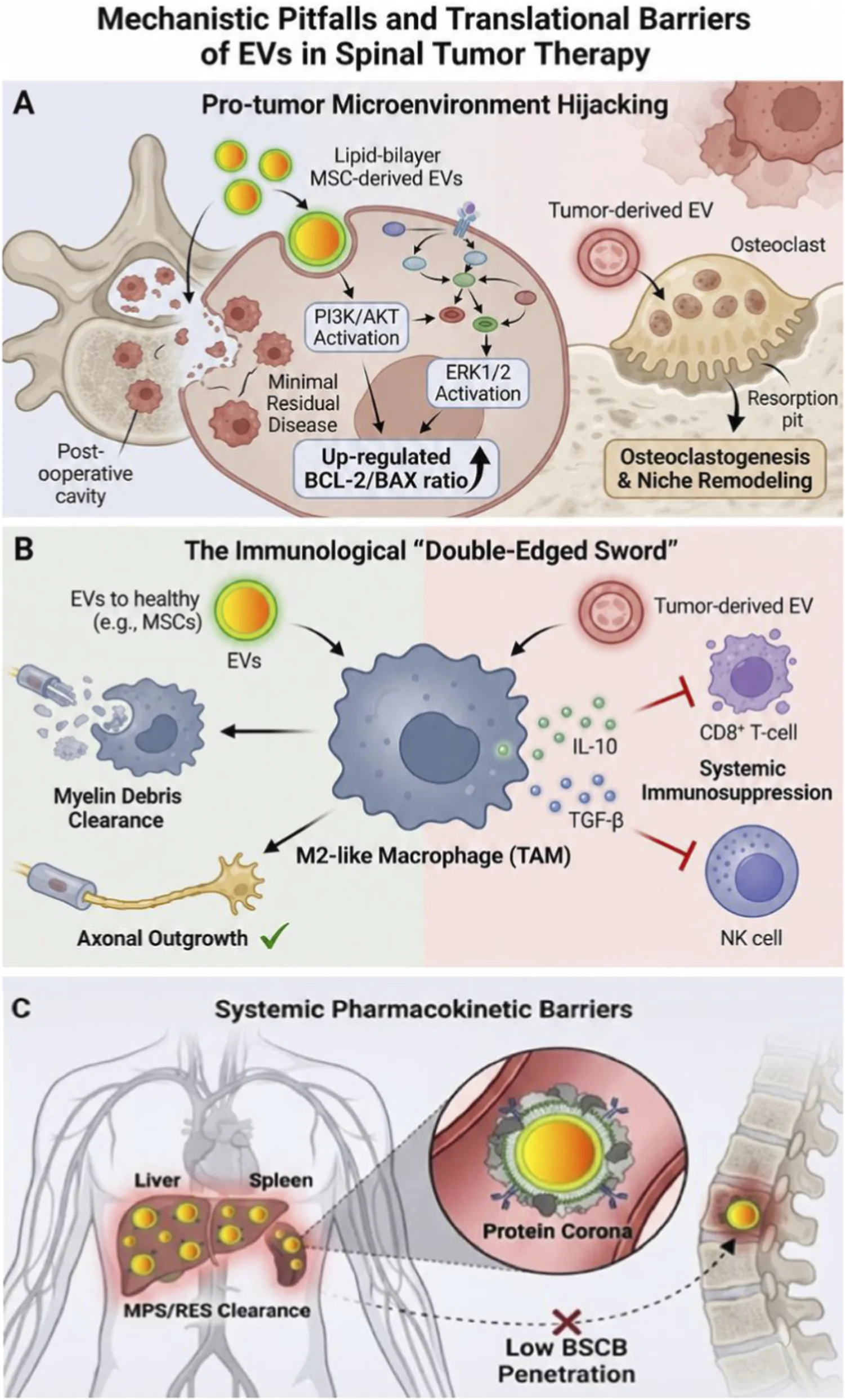
Mechanistic pitfalls and translational barriers of EVs in spinal tumor therapy.** (A)** Residual-tumor survival signaling and osteoclast-mediated niche remodeling. **(B)** M2-like tumor-associated macrophages may support myelin-debris clearance and axonal outgrowth but also systemic immunosuppression. **(C)** Mononuclear phagocyte system/reticuloendothelial system clearance, protein-corona formation and limited blood-spinal cord barrier penetration restrict systemic EV delivery.

EVs have potential as cell-free nanocarriers that mediate intercellular communication, cross the blood-spinal cord barrier (BSCB) and deliver therapeutic payloads. Their use in spinal tumors nevertheless carries important risks (Möller and Lobb, 2020). Evidence from isolated SCI or simplified tumor models may not capture the postoperative tumor–immune–inflammatory microenvironment (Pando et al., 2026). Source-cell characteristics and endogenous cargo can promote immune evasion or other pro-tumorigenic responses (Elfaki et al., 2024) Clinical translation therefore requires systematic profiling of recurrence risk, immunosuppression and off-target effects (Pathania et al., 2021). The principal biological risks and translational barriers are summarized in Figure 7.

### 6.1 The dual nature in microenvironmental modulation and pro-tumorigenic risks

In simple SCI animal models devoid of a neoplastic background, mesenchymal stem cell-derived EVs (MSC-EVs) exhibit remarkable regenerative potential. They remodel the microvascular network of the injured area and significantly drive axonal regeneration and remyelination by precisely delivering neurotrophic factors, pro-angiogenic molecules (e.g., VEGF, Ang-2), and specific non-coding RNAs (e.g., miR-126, miR-216a-5p) (Schepici et al., 2023). However, against the highly heterogeneous pathological backdrop of spinal tumors, this physiological mechanism intended for tissue repair faces a severe qualitative inversion. Due to the classical double-edged sword effect of MSC-EVs in tumor biology, their pro-regenerative properties are highly susceptible to being hijacked by the pathological microenvironment, thereby transforming into an auxiliary force that drives tumor progression (Zhang et al., 2025).

In clinical settings, minimal residual disease (MRD) commonly remains within the surgical cavity and surrounding cancellous bone following the decompression and resection of spinal tumors. For residual cancer cells in a dormant or early proliferative state, the pro-regenerative microenvironment fostered by MSC-EVs could inadvertently facilitate tumor recurrence. Studies have revealed a deep and complex vesicular communication network between tumor cells and MSCs. The abundance of pro-angiogenic factors and matrix-remodeling enzymes carried by MSC-EVs, while attempting to restore spinal cord perfusion, may inadvertently provide metabolic support to residual tumor clones, triggering the growth of tumor neovasculature (Easwaran et al., 2025).

For instance, emerging evidence indicates that MSC-EVs can robustly trigger PI3K/AKT and ERK1/2 signaling cascades within target cells (Easwaran et al., 2025). This process not only enhances cellular anti-apoptotic capacity (e.g., upregulating the BCL-2/BAX ratio) but also molecularly facilitates the distant colonization and drug-resistance evolution of solid tumors. Furthermore, within the bone marrow microenvironment, MSC-EVs can deliver long non-coding RNAs (e.g., lncRNA PVT1) that stabilize the expression of the oncogenic protein ERG by competitively sponging miR-183–5p, thereby promoting tumor cell proliferation and chemoresistance within the postoperative cavity (Zhao et al., 2019).

EV-mediated microenvironmental remodeling is a major driver of spinal metastasis, which most often originates from breast, prostate or lung cancer. Tumor-derived EVs can direct malignant-cell migration to bone and contribute to pre-metastatic niche formation (Cheng et al., 2023) (contextual review; outside Levels 1–5). For example, bone-tropic breast-cancer cells release miR-21-enriched vesicles that home to bone. After uptake by local osteoclast precursors, miR-21 represses PDCD4 and promotes osteoclastogenesis (Yuan et al., 2021) (Level 3 osseous-metastasis model). This shift disrupts osteoblast–osteoclast balance and creates an osteolytic niche that favors tumor infiltration, pathological fracture and secondary spinal-cord compression. In prostate-cancer models, EV-associated non-coding RNAs likewise remodel the bone microenvironment and contribute to immunosuppression (Duan et al., 2025) (contextual review; outside Levels 1–5).

Engineered tumor-derived EVs have been explored as homologous targeting vectors, but their safety boundaries remain uncertain. Because these vesicles may retain mutant proteins, oncogenic transcripts and pro-metastatic miRNAs from the parent cells, therapeutic loading can co-deliver endogenous pathogenic cargoes (Clancy and D’Souza-Schorey, 2023). Such cargoes could transform surrounding stromal cells, disrupt endothelial barriers or awaken dormant MRD. Until molecular detoxification and release criteria are established, the use of MSC-EVs or tumor-derived EVs in a tumor-residual spinal microenvironment should be considered biologically hazardous and translationally premature (Zhang et al., 2025).

### 6.2 Adverse immunomodulation and the attenuation of antitumor immunity

TA-SCI repair creates an immunological dilemma. Neural survival and regeneration benefit from controlled anti-inflammatory signaling, whereas MRD clearance requires preserved antitumor immunity. Without spatial and temporal control, EV-mediated immunomodulation may therefore produce unintended adverse effects (Fan et al., 2023).

In pure mechanical nerve injury without a neoplastic background, early suppression of inflammation is important for neuronal survival. MSC-EVs and M2 macrophage-derived EVs can reduce neurotoxic cytokines, including TNF-α, IL-1β and IL-6, through inhibition of the TLR4/MyD88/NF-κB cascade. They may consequently shift infiltrating macrophages and resident microglia from a pro-inflammatory M1 phenotype toward a tissue-reparative M2 phenotype (Luan et al., 2024; Jiang and Zhang, 2021; Shao et al., 2023) (Level 4). This polarization can facilitate myelin-debris clearance and limit reactive astrogliosis and glial-scar formation, thereby supporting neural-circuit reconstruction.

In patients with malignancy, however, an M2-like macrophage state overlaps with the phenotype of tumor-associated macrophages (TAMs) and can contribute to immune evasion (Yin and Wang, 2023). TAMs release IL-10, TGF-β and prostaglandins, which can impair cytotoxic T-lymphocyte and NK-cell function and recruit regulatory T cells and MDSCs. Tumor-derived EVs may reinforce this state through non-coding RNAs such as miR-21–5p, miR-155–5p and lncRNA HMMR-AS1. After uptake by macrophages, these cargoes can suppress PTEN or activate STAT3, PI3Kγ and Wnt/β-catenin signaling, thereby supporting invasion, metastasis and chemoresistance (Chen WX. et al., 2021; Wang et al., 2024).

This immunological paradox has direct safety implications. Strongly immunosuppressive MSC-EVs, if administered systemically or at high local doses during TA-SCI repair, may reinforce residual-tumor immunosuppression and weaken antitumor surveillance (Fan et al., 2023). The boundary between reparative M2 polarization and pro-tumorigenic TAM-like polarization must therefore be defined in immunocompetent, tumor-bearing spinal models. Precise spatiotemporal separation of neural repair from antitumor immunity, including through targeted EV engineering or hydrogel-based delivery, remains an unresolved safety requirement (Li et al., 2025).

### 6.3 Off-target delivery, biodistribution uncertainty, and long-term safety

A central unresolved issue is that the biodistribution of therapeutic EVs remains highly variable across EV sources, engineering strategies, administration routes, disease states, and host immune conditions.

Systemic administration is clinically convenient, but it does not ensure efficient delivery to spinal lesions (Gupta et al., 2023). EV pharmacokinetics and long-term tissue distribution vary with source, formulation and host state, creating substantial off-target uncertainty.

A primary hurdle for systemic EV administration is off-target accumulation followed by rapid clearance through the mononuclear phagocyte and reticuloendothelial systems. *In vivo* tracking studies indicate that many vesicles are captured by hepatic Kupffer cells, splenic macrophages and pulmonary capillaries soon after entering the circulation. This occurs even when they carry targeting peptides, aptamers or antibodies (Pavlova et al., 2026) (Gupta et al., 2023). The problem is compounded by the protein corona, a layer of opsonins, complement proteins and lipoproteins that adsorbs onto the EV surface in blood. The corona can mask engineered ligands, reduce lesion homing and accelerate innate-immune clearance (Liam-Or et al., 2024).

For spinal-tumor applications, off-target distribution is a major systemic safety concern. Cytotoxic drugs or gene-editing tools carried by EVs may damage the liver and spleen when vesicles accumulate in these organs (Zhang et al., 2023). Surface-modified EVs may also bind receptors on hepatic or splenic endothelial cells and macrophages, increasing receptor-mediated toxicity. In addition, endogenous tumor EVs can act as decoys that compete with therapeutic vesicles for uptake and protein-corona interactions, further reducing delivery efficiency (Liam-Or et al., 2024).

Because the liver and spleen act as systemic sinks, even tumor-targeted EVs may show low trans-BSCB delivery to deep spinal nerves and tumor foci. Escalating the injected dose to compensate for this loss could increase the risk of immune-mediated toxicity, including cytokine-release reactions (Gupta et al., 2023). Long-term EV distribution also remains incompletely characterized because non-invasive tracking methods that combine high spatial and temporal resolution are still limited (Pavlova et al., 2026). Biomimetic strategies such as CD47 display may reduce phagocytic clearance, but their safety and durability require further validation (Parada et al., 2021).

Because spinal tumor suppression and neural repair may require repeated or prolonged intervention, long-term safety remains a distinct unresolved issue. The favorable biocompatibility observed in single-dose studies may not reliably predict the consequences of chronic or repeated EV administration (Wiklander et al., 2019). Potential concerns include hepatic and splenic accumulation, macrophage saturation or exhaustion, neutralizing antibody formation, hypersensitivity, and altered clearance after repeated exposure (Zhu et al., 2017). For EVs carrying miRNAs or other gene-regulatory cargoes, prolonged exposure may also produce unintended epigenetic or transcriptomic effects in non-target tissues, with possible consequences for immune surveillance or dormant tumor control (O’Brien et al., 2020). Therefore, repeated-dose toxicology, long-term biodistribution, and toxicokinetic studies in immunocompetent, tumor-bearing spinal models are required before clinical translation can be responsibly considered.

### 6.4 Risk–benefit stratification and spatiotemporal decoupling

A clinically useful safety framework must stratify regenerative mechanisms by pathway and tumor type. PTEN inhibition or PI3K/AKT/mTOR activation has an established high-risk profile across breast, lung and prostate cancers, particularly in PIK3CA/AKT-driven breast cancer and PTEN-deficient prostate cancer (Khorasani et al., 2024; Sanaei et al., 2022; Cham et al., 2021). NGF/TrkA signaling has an intermediate, tumor-type-dependent risk: tumor-promoting effects are supported in breast and advanced prostate cancer, whereas evidence in lung cancer varies by histological subtype (Bruno et al., 2022; Di Donato et al., 2019; Gao et al., 2018). RhoA suppression by miR-133 b appears lower risk at the tumor-cell level, but its effects in stromal, endothelial, immune and neural compartments remain uncertain (Tsubaki et al., 2021; Liu et al., 2017; Hodge et al., 2003). Pro-angiogenic VEGF cargoes and sustained M2-like immunomodulation should be considered high risk during suspected MRD. Remyelination-directed cargoes without recognized proliferative signaling may be provisionally lower risk, but cannot be considered safe before tumor-bearing validation. Table 4 summarizes these evidence-based categories, neural-repair rationales and control strategies.

**TABLE 4:** Translational risk assessment of EV-based therapeutic strategies for spinal tumors.

Regenerative mechanism/representative EV strategy	Neural repair rationale	Breast cancer evidence	Lung cancer evidence	Prostate cancer evidence	Overall risk category	Recommended control strategy	References
PTEN inhibition–PI3K/AKT/mTOR activation (miR-26a-modified MSC-EVs; A2 astrocyte EV miR-5121)	Promotes axonal regeneration, BSCB repair and cell-survival signalling	Established: frequent pathway activation and clinically validated therapeutic targeting	Established: recurrent oncogenic activation linked to growth, invasion and drug resistance	Established: PTEN loss and AR–PI3K/AKT/mTOR crosstalk are major progression mechanisms	High	Avoid unrestricted systemic or prolonged exposure; require neural/endothelial recipient restriction, local finite dosing and documented tumour control	(Chen et al., 2021a; Wang et al., 2025; Khorasani et al., 2024; Sanaei et al., 2022; Cham et al., 2021)
NGF/TrkA activation (ADSC/UCMSC-EV miR-199a-3p or miR-145–5p)	Stimulates neurite outgrowth and axonal bridging	Established mechanistic concern: mitogenic, anti-apoptotic, angiogenic and invasion-associated effects	Controversial/subtype-dependent: strongest association in squamous cell carcinoma; limited functional and clinical evidence	Established mechanistic concern in advanced/CRPC models: proliferation, invasion and EMT	Intermediate; tumour-type dependent	Use local low-dose delivery only after MRD assessment; consider tumour-selective low-exposure TrkA inhibition and monitor neural and tumour pharmacodynamics in parallel	(Hwang et al., 2023; Bruno et al., 2022; Di Donato et al., 2019; Gao et al., 2018)
RhoA suppression (miR-133 b-modified MSC-EVs)	Relieves growth-cone inhibition and supports axonal extension	Relatively safe at the tumour-cell level: RhoA inhibition reduced metastatic behaviour in a breast cancer model	Relatively safe but incompletely defined: RhoA knockdown reduced proliferation and increased apoptosis in lung cancer cells	Relatively safe at the tumour-cell level: RhoA activity supports PC-3 invasion; stromal effects remain unresolved	Lower but context-dependent	Verify recipient-cell specificity and downstream ERK/STAT3/CREB activation; assess tumour, stromal, endothelial and immune compartments separately	(Li et al., 2018; Tsubaki et al., 2021; Liu et al., 2017; Hodge et al., 2003)
VEGF-mediated angiogenesis (NSC-EVs or proangiogenic MSC-EVs)	Restores vascular supply and supports neural tissue survival	Established concern when residual disease is present	Established concern when residual disease is present	Established concern, although the direction of EV-mediated angiogenesis is source-dependent	High during suspected MRD	Restrict to local, time-limited delivery after oncological control; measure tumour perfusion/microvessel density separately from functional vascular repair	(Sintakova and Romanyuk, 2024; Easwaran et al., 2025; Alcayaga-Miranda et al., 2016)
Sustained M2-like polarization/anti-inflammatory EV signalling	Reduces neuroinflammation, clears myelin debris and supports remyelination	Established concern: may reinforce TAM-mediated immune suppression	Established concern: may weaken antitumour immunity in residual disease	Established concern: may support an immunosuppressive, tumour-permissive niche	High during suspected MRD	Use transient or staged immunomodulation; preserve CTL/NK surveillance and distinguish reparative macrophages from persistent TAM-like states	(Schepici et al., 2023; Zhang et al., 2025; Yin and Wang, 2023)
Tbr1/Wnt-directed remyelination (hUCMSC-EV miR-23a-3p)	Promotes OPC differentiation and myelin-protein expression	Controversial/insufficient: Wnt effects are cell- and subtype-dependent; EV-specific tumour evidence is lacking	Controversial/insufficient: no direct spinal-metastasis evidence	Controversial/insufficient: no direct spinal-metastasis evidence	Provisionally lower; unproven	Do not label clinically safe; use local finite dosing and test tumour growth, OPC differentiation and remyelination together in tumour-bearing models	(Qin et al., 2024)

Risk mitigation should integrate pathway selection, dose, anatomical confinement and treatment phase. For mTOR- or TrkA-activating strategies, unrestricted systemic dosing and prolonged exposure should be avoided. Local administration of the lowest neurologically active EV dose could be combined with low-exposure inhibition directed preferentially at residual tumor cells. This combination may also attenuate neural repair and must therefore be evaluated using paired tumor and neural pharmacodynamic readouts. Temporal sequencing offers another approach: antitumor therapy should dominate the immediate MRD phase, whereas regenerative EVs should be introduced only after local control has been documented and should be delivered over a finite, prespecified period.

Stimuli-responsive platforms may help separate antitumor activity from neural repair in space and time. An ROS-responsive EV-integrated hydrogel achieved lesion-responsive release in experimental SCI (Cao et al., 2025) (Level 4). A pH-responsive hydrogel produced rapid aminoguanidine release followed by sustained EV release in a non-neoplastic injury model (Wang et al., 2023) (Level 4). Acidic-pH-responsive, integrin-targeted EVs also accelerated 5-fluorouracil release and antitumor activity in a tumor model (Kim et al., 2024) (non-spinal tumour model; Level 3 if *in vivo*). MMP-2- and MMP-9-sensitive hydrogels have enabled protease-responsive EV release in tissue-repair models (Meng et al., 2025; Zhou et al., 2019) (non-neoplastic tissue-repair models; outside the spinal-tumour hierarchy). A postoperative spinal-tumor depot could therefore be designed to release an antitumor payload first and reparative EVs only after a defined delay. However, ROS, acidosis and MMP activity occur in both residual tumors and healing tissue. A single trigger is not sufficiently specific; a safer design would combine microenvironmental sensing with a tumor- or neural-cell ligand, externally programmed timing or Boolean dual gating.

These strategies should be evaluated in immunocompetent orthotopic spinal-tumor models that incorporate surgical decompression and measurable residual disease. Tumor burden, local recurrence, angiogenesis, immune suppression, axonal regeneration, electrophysiological recovery, biodistribution, trigger thresholds, and release kinetics should be assessed in parallel. Only formulations that preserve neurological benefit without increasing residual-tumor growth or weakening antitumor immunity should advance toward repeated-dose and translational studies.

## 7 Translational challenges and barriers to clinical application

Moving from *in vitro* proof-of-concept studies to clinically authorized products requires solutions to major manufacturing, quality-control and regulatory challenges (Xu G. et al., 2025). EVs are heterogeneous assemblies of lipids, proteins and nucleic acids, and their production, characterization and batch stability are more complex than those of many small-molecule drugs or monoclonal antibodies (Carney et al., 2025). These barriers must be addressed through standardized processes and internationally aligned quality frameworks before clinical translation can proceed (Welsh et al., 2024).

### 7.1 Source selection, standardized isolation, and scalability

Currently, clinical translation of EVs faces a fundamental dilemma in cell source selection. Utilizing primary cells (e.g., MSCs) is plagued by replicative senescence and spontaneous phenotypic drift during *ex vivo* expansion, leading to drastic degradation in vesicle yield and molecular cargo across passages. This makes establishing a Good Manufacturing Practice (GMP)-compliant Master Cell Bank (MCB) exceedingly difficult (Tong et al., 2025). Conversely, employing immortalized cell lines engineered for consistency carries the grave ethical and biological risk of introducing oncogenic mutant sequences or viral remnants into patients with spinal tumors, prompting exceptionally high regulatory hurdles (Tong et al., 2025).

Regarding the industrialization of isolation and purification technologies, bench-top research still relies heavily on traditional ultracentrifugation (UC). However, UC is characterized by extremely low throughput, time-consuming protocols, and a high propensity for protein aggregate co-sedimentation, rendering this crude process entirely inadequate for handling large volumes of conditioned media in clinical trials (Visan et al., 2022). The industry urgently requires a paradigm shift toward coupled processes like Tangential Flow Filtration (TFF) and Size Exclusion Chromatography (SEC). TFF efficiently and gently concentrates large media volumes, while the subsequent SEC step elegantly depletes residual soluble protein impurities and free nucleic acid fragments (Kim et al., 2025). The TFF-SEC tandem process not only enhances overall recovery but also maximally preserves the bioactivity of vesicular surface receptors, improving the core purity metric of particle-to-protein ratio (Visan et al., 2022).

After purification, EV preparations must be characterized according to the multidimensional standards of MISEV 2023. The framework requires documentation of non-vesicular particles and soluble co-isolated impurities, including abundant serum proteins such as albumin. Comparative analysis of positive and negative markers is needed to determine whether a biological effect is attributable to EVs or to co-isolated contaminants (Welsh et al., 2024).

### 7.2 Cargo loading, release characterization, and manufacturing reproducibility

EV therapies also face substantial challenges in loading exogenous drugs and nucleic acids (Dieu et al., 2025). Passive co-incubation is gentle but often produces insufficient intravesicular payload. Active methods such as electroporation and sonication can increase loading, but may disrupt the membrane and promote aggregation during reassembly. These changes can cause premature leakage, alter surface properties and accelerate clearance of damaged vesicles by the mononuclear phagocyte system (Gong et al., 2025; Dieu et al., 2025).

Manufacturing reproducibility is particularly important because donor-cell source, passage number, culture conditions, isolation method and storage can alter EV yield, cargo composition, surface markers and potency. Release kinetics and batch-to-batch variation also remain difficult to control. After uptake, therapeutic activity may be limited by inefficient endosomal escape: a substantial fraction of EVs enters the endolysosomal pathway, where acidic hydrolysis can degrade internal RNA before cytosolic delivery (Hagedorn et al., 2024). Small changes in the culture environment can further alter EV composition and loading capacity (Rayamajhi et al., 2023). A rigorous Chemistry, Manufacturing and Controls (CMC) framework is therefore required for standardized EV products (Xu G. et al., 2025).

### 7.3 Route of administration and storage stability

The administration route must balance access to spinal lesions against systemic exposure. Intravenous delivery is limited by off-target distribution and by anatomical barriers including the BSCB and dura mater (Gupta et al., 2023). Local EV-hydrogel implantation may reduce systemic concentration fluctuations, provide structural support and sustain release at the lesion (Wu et al., 2026). In a postoperative cavity containing MRD, however, scaffold degradation and release kinetics must be tightly controlled because prolonged regenerative signaling could support local invasion or recurrence (Chen et al., 2026).

Beyond anatomical barriers, storage conditions create additional logistical challenges. Most current EV formulations require an ultra-low-temperature (−80 °C) cold chain. Repeated freeze–thaw cycles can damage the lipid bilayer and cause payload leakage (Wang et al., 2026). Lyophilization with optimized cryoprotectants such as trehalose or mannitol may improve room-temperature or refrigerated stability, but the process must preserve surface receptors and nucleic-acid integrity (Wang et al., 2026).

### 7.4 Dose standardization, potency assays, and regulatory frameworks

Dose standardization remains a major regulatory barrier. For heterogeneous EV preparations with broad size distributions, no consensus defines an effective therapeutic dose (Jay, 2025). Nanoparticle-tracking analysis and BCA-based protein quantification can be confounded by non-vesicular lipoproteins and free proteins such as serum albumin, leading to overestimation of active vesicle numbers (Kim et al., 2025). Consistent with MISEV 2023, future trials should use orthogonal measurements, including particle-to-protein and protein-to-lipid ratios, to improve dose precision, inter-batch comparability and traceability (Welsh et al., 2024).

Regulatory compliance also requires validated *in vitro* potency assays. These assays should predict clinically relevant EV activity with acceptable reproducibility. In spinal tumors with concomitant neural injury, biochemical measurements alone are insufficient. Functional panels should therefore assess macrophage polarization, T-cell suppression and angiogenic activity, together with neural-repair readouts (Xu G. et al., 2025). Without such assays, EV candidates will have difficulty meeting regulatory and commercial requirements (Tertel et al., 2026).

In summary, EV therapeutics remain at a transitional preclinical stage. Clinical translation will require standardized production, validated potency assays and rigorous safety evaluation.

## 8 Future perspectives and concluding remarks

EVs combine biological-barrier transport, biocompatibility and engineering flexibility, making them candidate adjuncts for antitumor delivery and neural repair. Current evidence nevertheless relies largely on primary-tumor or non-neoplastic SCI models. These studies provide mechanistic and engineering guidance but do not establish efficacy or safety in spinal tumors.

Clinical translation requires progress in three areas. First, composite models should reproduce tumor compression, surgical decompression and postoperative MRD so that sEV efficacy and safety can be evaluated in a representative spinal microenvironment. Second, pathway- and tumor-specific safety boundaries must be defined because regenerative signaling may also support residual disease. Risk-adapted dosing and staged local delivery should therefore be evaluated rather than assuming that regenerative signalling is intrinsically safe. ROS-, pH- and MMP-responsive systems may provide trigger-dependent release, but the same cues occur in postoperative injury and residual tumors. Dual-gated targeting, predefined release kinetics and parallel monitoring of tumor control and neurological repair are therefore required (Cao et al., 2025; Wang et al., 2023; Kim et al., 2024; Meng et al., 2025; Zhou et al., 2019). Third, clinical development requires scalable GMP-compliant production and robust potency assays aligned with current international guidance, including MISEV 2023.

In conclusion, sEVs remain an early-stage strategy for the dual challenge of spinal tumor control and neural repair. Their future value will depend on moving from idealized surrogate models toward clinically relevant spinal tumor models, while prioritizing safety boundaries, pharmacokinetic profiling, and translational restraint.
